# Chemoenzymatic Synthesis and Purification of Bioorthogonally Tagged UDP‐GlcNAc and UDP‐GalNAc Analogues

**DOI:** 10.1002/cpz1.70277

**Published:** 2025-12-17

**Authors:** Ganka Bineva‐Todd, Benjamin Schumann

**Affiliations:** ^1^ Chemical Glycobiology Laboratory The Francis Crick Institute London UK; ^2^ Department of Chemistry Imperial College London London UK; ^3^ Faculty of Chemistry and Food Chemistry TUD Dresden University of Technology Dresden Germany; ^4^ Both authors contributed equally.

**Keywords:** bioorthogonal, chemoenzymatic synthesis, glycosylation, nucleotide sugar, pyrophosphorylase

## Abstract

Nucleotide‐sugar donors containing bioorthogonal moieties are important tools to study cellular glycosylation. Typically, the acetamide moiety in *N*‐acetylhexosamines such as GlcNAc and GalNAc is replaced by an acylamide with a clickable tag and converted to the corresponding uridine diphosphate analogue. These probes can then be tested for acceptance by glycosyltransferase enzymes *in vitro*. Lengthy procedures in synthetic chemistry currently limit the availability of bioorthogonal uridine diphosphate (UDP)‐sugar analogues. Chemoenzymatic synthesis has proven to be a powerful and effective alternative, and multiple approaches have been published to date. In this protocol, we describe a streamlined method for the generation of bioorthogonal UDP‐GlcNAc and UDP‐GalNAc analogues. We describe the chemical modification of d‐glucosamine and D‐galactosamine to incorporate bioorthogonal acylamides, the subsequent one‐pot multienzyme conversion to the corresponding UDP‐sugar analogues, and reproducible purification. Our approach features the bacterial kinase NahK and human pyrophosphorylase AGX1 as well as a recombinantly expressed AGX1 variant with an expanded substrate profile. The approach further features an inorganic pyrophosphatase and an alkaline phosphatase to improve enzymatic turnover and aid the purification process, respectively. The use of biosynthetic enzymes with substrate promiscuity extends the scope of bioorthogonal nucleotide‐sugar analogue structures to aid efforts in chemical glycobiology. © 2025 The Author(s). *Current Protocols* published by Wiley Periodicals LLC.

**Basic Protocol 1**: Chemical synthesis of bioorthogonally tagged acylamide analogues of D‐GlcNAc and D‐GalNAc

**Alternate Protocol 1**: Chemical synthesis of bioorthogonally tagged acylamide analogues of D‐GlcNAc and D‐GalNAc from a protected GlcNH_2_ or GalNH_2_ precursor

**Basic Protocol 2**: Conversion of D‐GlcNAc or D‐GalNAc analogues to UDP‐sugars using analytical‐ (reaction scouting) and preparative‐scale enzymatic synthesis and purification

## INTRODUCTION

Glycosylation is one of the most abundant post‐translational modifications, having profound importance in a wide range of biological processes (Gagneux et al., 2022; Varki, 2017). However, despite the importance in physiology, research into glycans is hampered by their nontemplated biosynthesis. Instead, glycans are biosynthesized using a number of glycosyltransferases in a combinatorial fashion. Glycosyltransferases use activated sugars as building blocks, often in the form of nucleotide‐sugars, to catalyze the formation of the glycosidic bond between the sugar and substrate nucleophile in a regio‐ and stereospecific manner (Rini JM, 2022; Schjoldager et al., 2020).

Chemically modified sugars have become invaluable tools to study glycosylation. The first analogues of D‐*N*‐acetylglucosamine (D‐GlcNAc) and D‐*N*‐acetylgalactosamine (D‐GalNAc) containing azide‐substituted acylamides were employed to study incorporation into cellular glycoproteins (Saxon et al., 2002; Vocadlo et al., 2003; Hang et al., 2003). Other analogues have been produced since, yielding acylamides of different sizes and with various bioorthogonal tags (Choi et al., 2019; Cioce et al., 2022; Debets et al., 2020; Zaro et al., 2011). Cellular glycosylation was probed using bioorthogonal chemistry, initially through the Staudinger–Bertozzi ligation and subsequently through variations of the 1,3‐dipolar cycloaddition, among others (Kufleitner et al., 2022; Parker & Pratt, 2020; Saxon et al., 2002). The assumption was that these sugars would be converted to the corresponding activated uridine diphosphate (UDP) derivatives by cellular biosynthetic pathways to then be used by glycosyltransferases. While true for sugar analogues with relatively small acylamides, larger analogues can be challenging to deliver to cells and enter native UDP‐sugar biosynthetic pathways. Differences in cellular sugar incorporation have been observed between cell lines of different organisms, and between compounds of different sizes (Batt et al., 2017; Kufleitner et al., 2024; Mukherjee et al., 2024; Tan et al., 2018). These findings prompted us and others to investigate the syntheses of UDP‐sugar analogues to assess their acceptance by biosynthetic enzymes and glycosyltransferases (Cioce et al., 2022; De León González et al., 2024; Liu et al., 2024).

**Figure 1:**
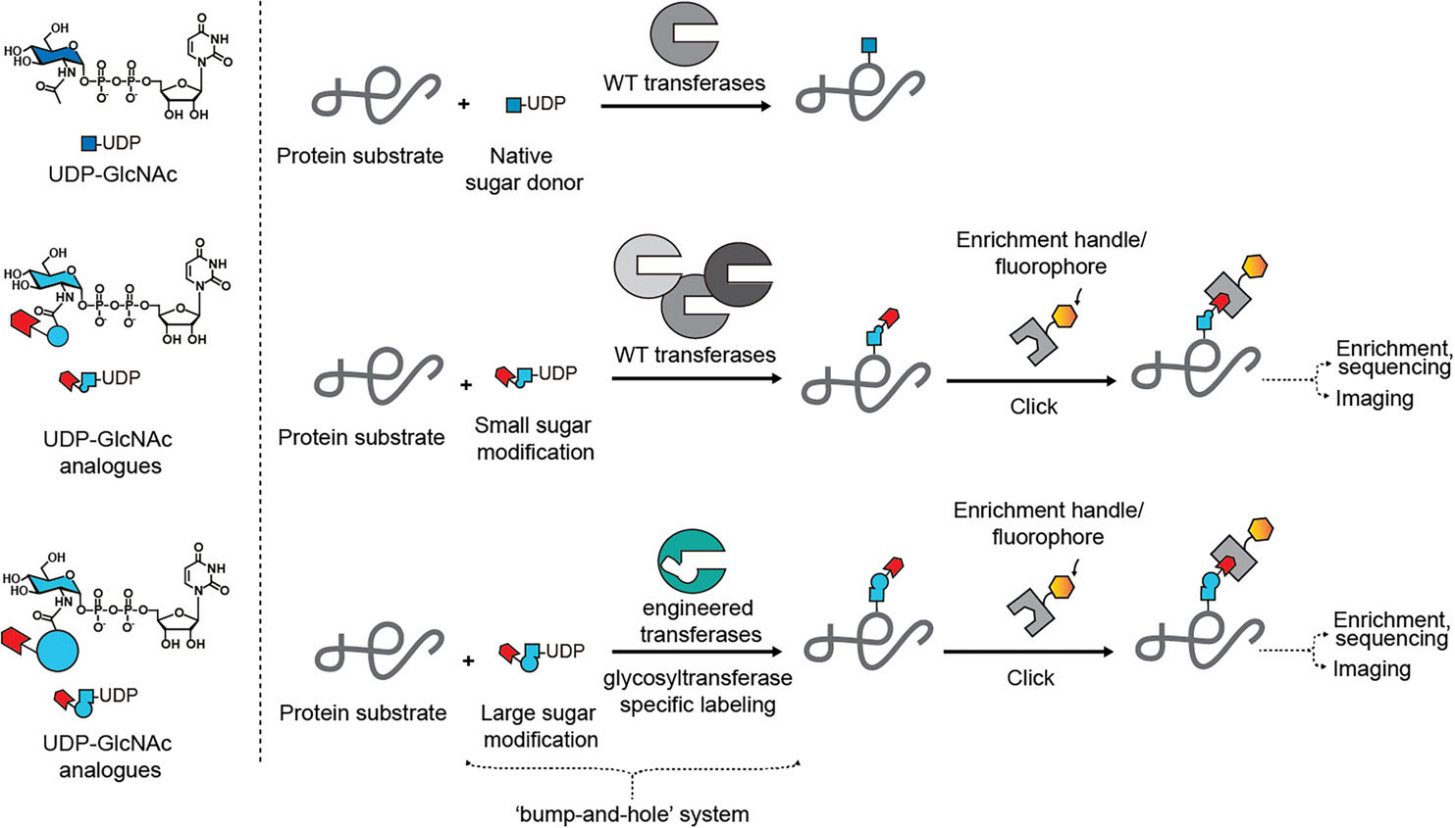
Applications of bioorthogonal UDP‐GlcNAc analogues as pan‐transferase and enzyme‐specific reporters. A rationally designed “hole” in the active site of the glycosyltransferase of interest paired with a “bumped” nucleotide‐sugar analogue accepted by the engineered enzyme allows for enzyme‐specific downstream tracking. Note that some larger modifications are accepted by wildtype glycosyltransferases (Tan et al., 2018; Kufleitner et al., 2024)

In addition to the necessity of sugar analogues to be converted to the corresponding nucleotide‐sugars, incorporation into the proteome also relies on their uptake by glycosyltransferases. Relatively small modifications are often taken up by wild‐type glycosyltransferases. In contrast, larger and branched acylamides are particularly suitable for a tactic termed “bump‐and‐hole engineering” in which the active site is enlarged to accommodate such modifications (Choi et al., 2019; Cioce et al., 2022; Debets et al., 2020; Liu et al., 2024). The new orthogonal enzyme–substrate pair can be used as an enzyme‐specific reporter system. Conjugation to fluorophores or biotin via a bioorthogonal tag enables visualization and/or enrichment for substrate profiling and glycosite mapping (Fig. 1).

Chemical nucleotide‐sugar syntheses have been used for decades, usually employing classical phosphate coupling chemistry (Ahmadipour & Miller, 2017; Beahm et al., 2014; Choi et al., 2019; Öhrlein, 2000; Tsukamoto & Kahne, 2011; Wagner et al., 2009). Synthetic routes require multistep synthesis procedures and multiple purifications that are well established, yet time‐ and resource‐intensive. Enzymatic *in vitro* syntheses have been gaining popularity in recent years (De León González et al., 2024; Liu et al., 2024; Wen et al., 2018), allowing for more time‐efficient and potentially greener routes of obtaining nucleotide‐sugar analogues. These efforts traditionally relied on native biosynthetic enzymes. In mammalian cells, UDP‐sugars can be generated via either *de novo* or salvage pathways. The UDP‐GalNAc salvage pathway starts with phosphorylation of GalNAc to GalNAc‐1‐phosphate by the kinase GALK2. This is followed by the condensation with UDP catalyzed by the pyrophosphorylase AGX1 that produces the nucleotide‐sugar and pyrophosphate (Boyce et al., 2011; Chuh et al., 2014; Freeze, 2022). Both GALK2 and AGX1 can be used to produce UDP‐GalNAc and analogues (Bourgeaux et al., 2005; Pouilly et al., 2012). A downside of both enzymes is their limited scope toward unnatural substrates with modified acylamides. The bacterial kinase NahK from *Bifidobacterium longum* is widely promiscuous, converting a large range of GalNAc analogues with modified acylamides to sugar‐1‐phosphates in good to quantitative conversion (De León González et al., 2024; Keenan et al., 2020; Li et al., 2011, Wen et al., 2018). Human AGX1 accepts a limited scope of small GalNAc‐1‐phosphate analogues *in vitro*, but larger GalNAc modifications or those with branched substituents are not well tolerated (Cioce et al., 2021; Wen et al., 2018; Zafar et al., 2024).

Similar to UDP‐GalNAc analogues, UDP‐GlcNAc analogues have been generated through the coupled activities of a kinase and a pyrophosphorylase (Chuh et al., 2014). These activities are somewhat less biomimetic since the mammalian GlcNAc salvage pathway does not directly phosphorylate the anomeric position. Rather, the 6‐position is first phosphorylated in cells to initially activate GlcNAc (Akella et al., 2019; Freeze, 2022). NahK is often employed to generate bioorthogonal GlcNAc‐1‐phosphate analogues (Liu et al., 2024; Wen et al., 2018). The bacterial enzyme GlmU from *Escherichia coli* is reported to be more promiscuous toward GlcNAc‐1‐phosphate analogues than AGX1 (Wen et al., 2018). GlmU has also been shown to tolerate a broader range of acylamide substituents in GlcNAc‐1‐phosphates than in GalNAc‐1‐phosphates. However, neither pyrophosphorylase has been able to accommodate GlcNAc‐1‐phosphate analogues modified with larger or branched acylamides (Wen et al., 2018). Interestingly, insertion of a linker between acylamide and modification has been found to enable acceptance of GlcNAc‐1‐phosphate by AGX1 (Tan et al., 2018; Kufleitner et al., 2024).

To expand the range of UDP‐sugar analogues that can be obtained *in vitro* or in cells, Kohler and colleagues engineered AGX1 in a structure‐guided fashion to accept chemically modified GlcNAc‐1‐phosphate analogues (Yu et al., 2012). Mutating the bulky hydrophobic Phe383 residue in the active site generated space for chemical modifications. Since then, both AGX1 variants F383G and F383A have been used (Mukherjee et al., 2024; Tarbet et al., 2018; Toleman et al., 2018; Wu et al., 2022; Cioce et al., 2021; Schumann et al., 2020; Zafar et al., 2024). The combination of NahK and AGX1^F383A^ has proven a reliable method for the generation of a wide variety of bioorthogonally tagged UDP‐GalNAc and UDP‐GlcNAc analogues *in vitro* (de León González et al., 2024; Liu et al., 2024).

While the enzymatic approach for the synthesis of sugar nucleotides has many advantages over the traditional synthetic route, the isolation of the enzymatic products is more challenging. The presence of ATP, UTP, and ADP in the reaction mixture, compounds with similar molecular weights and chemical properties as the desired products, precludes the use of a single chromatographic technique. Several groups have reported sequential purification by strong anion exchange (SAX) HPLC and C18 HPLC with ion‐pairing buffers (Ahmadipour et al., 2019; Wagstaff et al., 2015). Wen and colleagues (Wen et al., 2018) used silver nitrate or alkaline phosphatase to first remove the excess nucleotides and then obtain the product by P‐2 gel chromatography, while others used a combination of SAX and size exclusion after enzymatic digestion (Burkart et al., 2000; Frohnmeyer et al., 2024).

**Figure 2:**
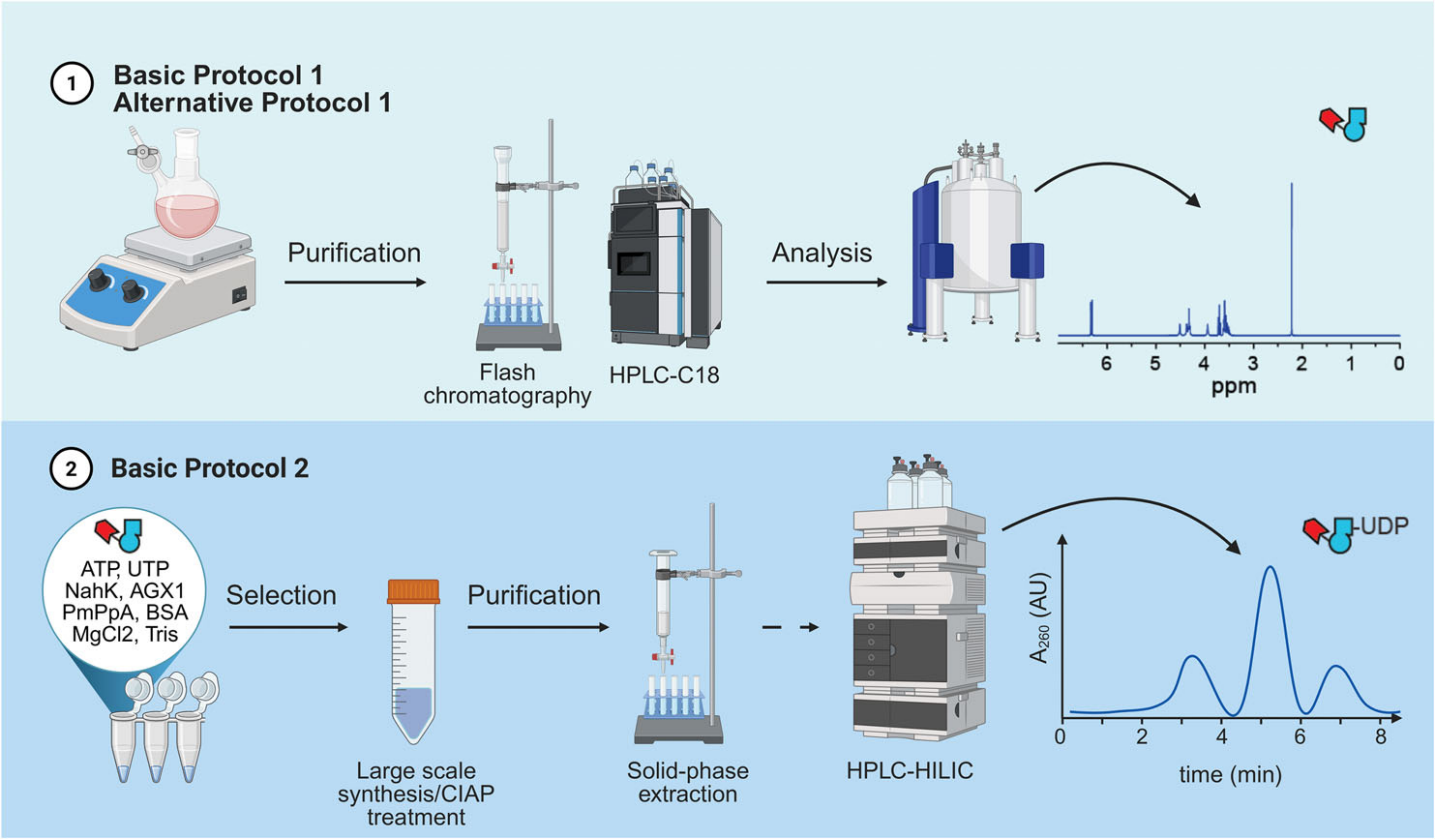
Flowchart illustrating each step of the synthesis. Created in BioRender. Bineva‐Todd, G. (2025) https://BioRender.com/i8krfxt.

**Figure 3:**
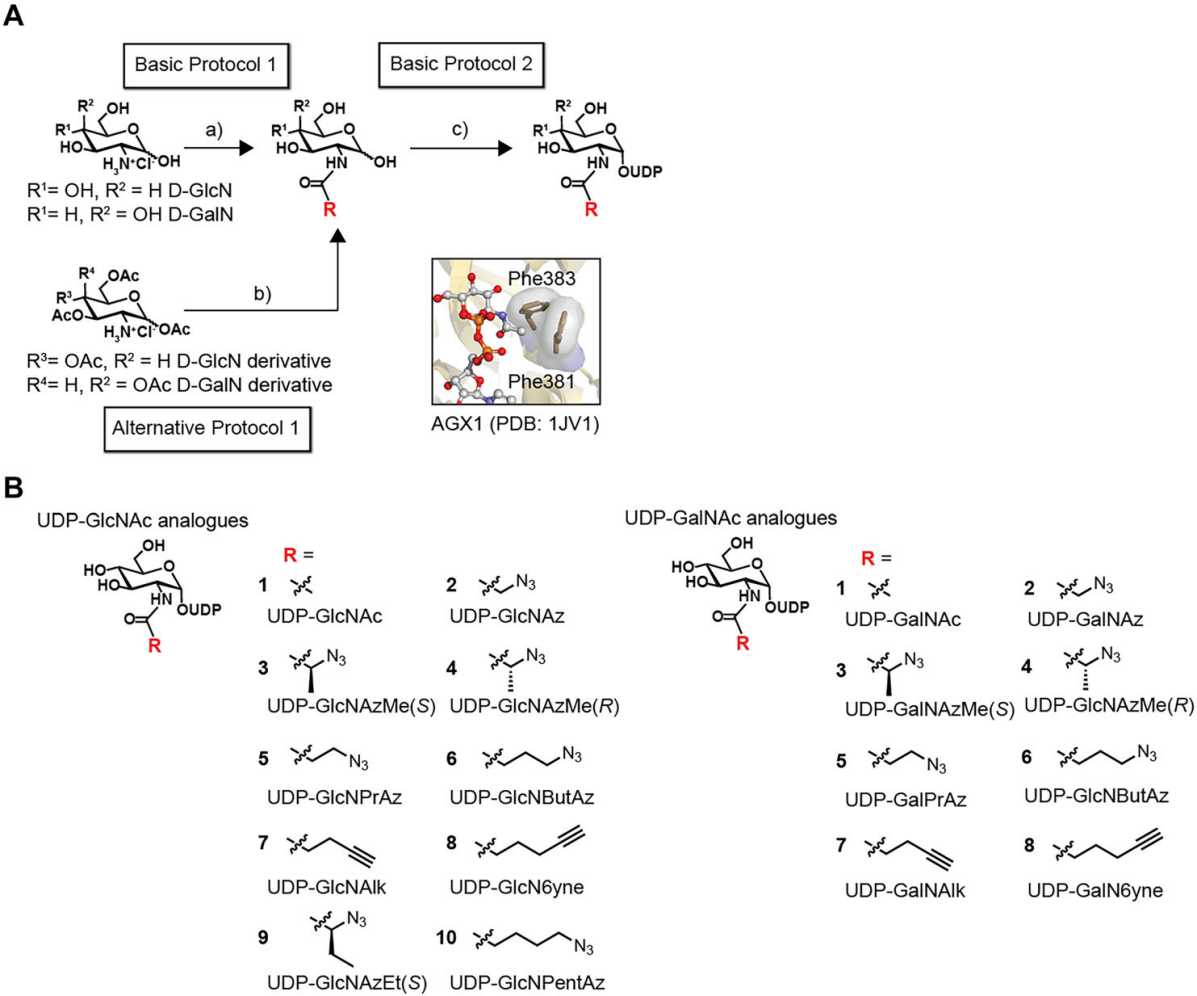
Synthesis of biorthogonal UDP‐GlcNAc and UDP‐GalNAc analogues. **(A)** Synthetic scheme including basic and alternative protocols: a) RCOOH, Et_3_N, EDC, HOBT, and MeOH, room temperature (RT) 3–16 h; b) RCOOH, DIPEA, COMU, and DMF, RT, 3–16 h, and then NaOMe and MeOH, RT, 2–5 h; c) NahK, AXG1, PmPpA, ATP, UTP, Tris·HCl pH 8, and MgCl_2_, 37°C, 16 h; Insert: crystal structure of human AGX1 with UDP‐GlcNAc (PDB 1JV1). **(B)** Examples of UDP‐GlcNAc and UDP‐GalNAc analogues.

In this protocol, we provide a step‐by‐step guide to demonstrate the use of an expanded chemoenzymatic synthesis method on both analytical and preparative scales. An optimized purification procedure is presented, employing a combination of enzymatic digestion and HILIC chromatography to reliably obtain bioorthogonally tagged UDP‐sugars in reasonable time, yield, and purity. An overview of each step is illustrated in Figure 2. The first basic protocol describes the chemical synthesis of GlcNAc and GalNAc analogues from commercial D‐hexosamine hydrochlorides and bioorthogonal carboxylic acids. The alternative method features the synthesis of GlcNAc and GalNAc analogues starting from protected precursors of D‐glucosamine and D‐galactosamine. The second basic protocol describes the analytical‐scale one‐pot chemoenzymatic synthesis of each nucleotide‐sugar analogue (Fig. 3). Analytical‐scale reaction scouting is necessary for optimizing the preparative‐scale enzymatic synthesis, also detailed in this protocol. We conclude with details of an optimized protocol for isolating the pure product from the complex mixture of compounds with similar chemical properties.

## Basic Protocol 1 CHEMICAL SYNTHESIS OF BIOORTHOGONALLY TAGGED ACYLAMIDE ANALOGUES OF D‐GlcNAc AND D‐GalNAc

Prior to the generation of UDP‐GlcNAc/GalNAc analogues, D‐GlcNAc and D‐GalNAc analogues are chemically synthesized. Analogues are generated by amide bond coupling between D‐galactosamine hydrochloride or D‐glucosamine hydrochloride and the respective carboxylic acids containing a bioorthogonal tag. The resulting D‐GalNAc and D‐GlcNAc analogues are purified from the reaction mixture by normal‐ and/or reverse‐phase chromatography to yield pure product ready for enzymatic conversion to the corresponding UDP‐GlcNAc or UDP‐GalNAc analogues. The purity of the product is confirmed by 1‐ and 2‐dimensional NMR.

*CAUTION*: All reactions must be carried out in a suitable, well‐ventilated fume hood. Lab coats, protective gloves, and safety goggles must be worn when handling reagents and reaction mixtures.

*CAUTION*: Always use a secondary container to hold the reaction vessel. The secondary container should be able to hold the entire contents of the primary container plus an extra 10% in volume.

### Materials

D‐(+)‐glucosamine hydrochloride (Sigma‐Aldrich, cat. no. G4875, CAS No: 66‐84‐2, 100 mg per analogue)
D‐ (+)‐Galactosamine hydrochloride (Sigma‐Aldrich, cat. no. G0500, CAS No: 1772‐03‐8, 100 mg per analogue)
Methanol, ≥ 99.8 %, anhydrous (Sigma‐Aldrich, cat. no. 5.89596, CAS No: 67‐56‐1, 5 ml per analogue)
Triethylamine, ≥ 99.5 % (Sigma‐Aldrich, cat. no. 471283, CAS No: 121‐44‐8, 175 µl per analogue)
Azido or alkyne‐containing carboxylic acids (4‐azidobutyric acid, Sigma‐Aldrich, cat. no. SY3H98B927CF, CAS No: 54447‐68‐6)
5‐hexynoic acid (Sigma‐Aldrich, cat. no. 544000, CAS No: 53293‐ 00‐8, 1.1–2.2 mmol per analogue)
*N*‐(3‐dimethylaminopropyl)‐*N*′‐ethylcarbodiimide (EDC; Sigma‐Aldrich, cat. no. 39391, CAS No: 1892‐57‐5, 195 mg per analogue)
1‐hydroxybenzotriazole hydrate (HOBT), ≥97.0% dry, peptide synthesis grade (Sigma‐Aldrich, cat. no. 54802, CAS No: 123333‐53‐9, 76 mg per analogue)
Dichloromethane, HPLC grade (Fisher Scientific, cat. no. 10343602, CAS No: 75‐09‐2, 200–500 ml)
Methanol, HPLC grade (Sigma‐Aldrich, cat. no. 34860, CAS No: 67‐56‐1, 20–100 ml)
3‐methoxyphenol, 96% (Sigma‐Aldrich, cat. no. 328456, CAS No: 150‐19‐6)
Sulfuric acid (Sigma‐Aldrich, cat. no. 339741, CAS No: 7664‐93‐9)
Ethanol (Sigma‐Aldrich, cat. no. 493546, CAS No: 64‐17‐5)
Insolute HM‐N (Biotage, cat. no. 9800‐0500)
Silica (Sigma‐Aldrich, cat. no. 1.07734, CAS No: 7631‐86‐9)
Formic acid, Optima LC‐MS grade (Fisher Scientific, cat. no. A117‐50)
Methanol‐d4 (Sigma‐Aldrich, cat. no. 441384, CAS No: 811‐98‐3)
Acetonitrile, HPLC grade (Sigma‐Aldrich, cat. no. 34851, CAS No: 75‐05‐8)
Fume hood with suitable ventilation

Schlenk line connected to N_2_ supply
Round‐bottom flasks (25, 100, and 250 ml)
Syringes
Rubber stoppers
Magnetic plate stirrer (Heidolph MR Hei‐Tec or equivalent)
Magnetic stirrer bar
TLC plates (Macherey‐Nagel, DC‐Fertigfolie Polygram SIL G/UV254, 0.2 mm thickness or equivalent)
Heating gun (Bosch 230 V, 50 Hz, 1800 W or equivalent)
Ultra‐performance liquid chromatography‐mass spectrometry (UPLC‐MS) or liquid chromatography‐mass spectrometry (LC‐MS) instrument (Acquity UPLC‐MS (Waters) equipped with ACQUITY UPLC BEH C18 column or similar) or access to MS facilities
Rotary evaporator (Büchi 300 or equivalent)
Flash purification system (Isolera One Flash Chromatography instrument with Sfär Silica D Duo preloaded cartridges or glassware and equipment for manual flash chromatography)
Solid phase extraction cartridge (Sfär Silica D Duo 25 g cartridge, Biotage; Sep‐Pak C18 20 cc Vac Cartridge, 5 g, Waters; or equivalent)
High‐performance liquid chromatography purification system [Agilent 1260 Infinity II MDAP system (Agilent Technologies) equipped with a 5 Prep‐C18 (100Å, 5 µm, 21.2 mm × 50 mm) column or equivalent]
Nuclear magnetic resonance instrument (NMR; Bruker Avance‐400 MHz or equivalent)

*CAUTION*: Methanol is toxic. Use a Schlenk line with a bubbler to allow any generated gas to escape. HOBT is a desensitized explosive and eye irritant. Weigh out and handle in a fume hood with caution. Dichloromethane is an irritant and carcinogenic. 3‐methoxyphenol is toxic and an irritant, sulfuric acid is corrosive, and ethanol is flammable and an irritant. Acetonitrile is an irritant and toxic and should be handled with care. These reagents should be handled in a fume hood with care. The automated flash chromatography system or manually packed column should be placed in a fume hood or ventilated cabinet. Wear personal protection equipment while handling all these reagents and while operating the flash chromatography system due to the hazardous nature of the solvents and the potential risk of spillage from an instrument operating at medium pressure.

#### Synthesis

1 Weigh out D‐glucosamine or D‐galactosamine hydrochloride (100 mg, 0.47 mmol) and transfer it into a flame‐dried, 25‐ml round‐bottom flask equipped with a magnetic stirrer bar. Close the flask with a rubber stopper and connect it to a Schlenk line. Open the N_2_ line and let the gas flow into the flask. Transfer anhydrous methanol (5 ml) into the flask using a syringe and needle and start stirring by positioning the flask above a magnetic plate stirrer (150–200 rpm is sufficient).
  D‐glucosamine hydrochloride and D‐galactosamine hydrochloride form white suspensions in methanol. Adding the base in step 2 will deprotonate the salt, and the suspension will turn into a colorless solution.
2 Measure triethylamine (175 µl, 1.25 mmol, 2.67 eq.) with a syringe and needle. Add it to the suspension by inserting the needle into the rubber septum and dispensing the contents of the syringe. Leave to stir on the magnetic plate stirrer for 5 min.
  At this step, the reaction mixture will become a colorless solution.
  The reaction is not highly sensitive to water; therefore, the use of anhydrous triethylamine is not necessary.
3 Add the carboxylic acid containing a bioorthogonal tag (1.1 mmol, 2.4 eq.) with a syringe and needle without opening the reaction flask. Cool the solution to 0 °C by placing the flask in an ice‐filled container while continuing to stir, and keep under an inert atmosphere for a further 10 min.
4 Remove the septum and quickly add EDC (195 mg, 1.25 mmol, 2.67 eq.) and HOBT (76 mg, 0.56 mmol, 1.2 eq.) to the reaction mixture and warm to room temperature by removing the ice bath.
  On adding the activators, the solution will become dark yellow to brown in color.
  The reaction is not air sensitive and removing the septum to add the solid reagents for a short time should not have an effect.
5 Leave the reaction mixture to stir for 3 to 16 h at room temperature.
  The reaction may be completed in less than or more than 16 h. Start monitoring the reaction from 3 h onward using TLC and/or LC‐MS (optional).
  If the starting material (SM) is still present after TLC, add more of the acid (1.2 eq.), EDC (1.3 eq.), and HOBT (0.6 eq.) and leave to stir until all SM is consumed.
6 Monitor the reaction using TLC and/or LC‐MS.
  Monitoring the reaction by LC‐MS is an alternative method.

#### Reaction monitoring by TLC

Solvent mixture: dichloromethane: methanol 9:1 (v/v)

“Sugar stain”: 0.1% (v/v) 3‐methoxyphenol and 2.5% (v/v) sulfuric acid in ethanol (see recipe)

With a pencil, draw a horizontal line near the bottom of the TLC plate. Spot separately the following samples along this line: (A) starting material (SM); (B) co‐spot (with both SM and reaction mixture); (C) reaction mixture. Develop the plate by placing it in a TLC chamber with 9:1 dichloromethane: methanol (v/v) and running it until the solvent line is close to the top of the TLC plate.

The solvent line in the TLC chamber should be below the spots.

Leave the TLC plate to dry for a few minutes in a fume hood. Dip it briefly in sugar stain and heat with a heating gun until dark yellow/brown spots appear.

**Figure 4:**
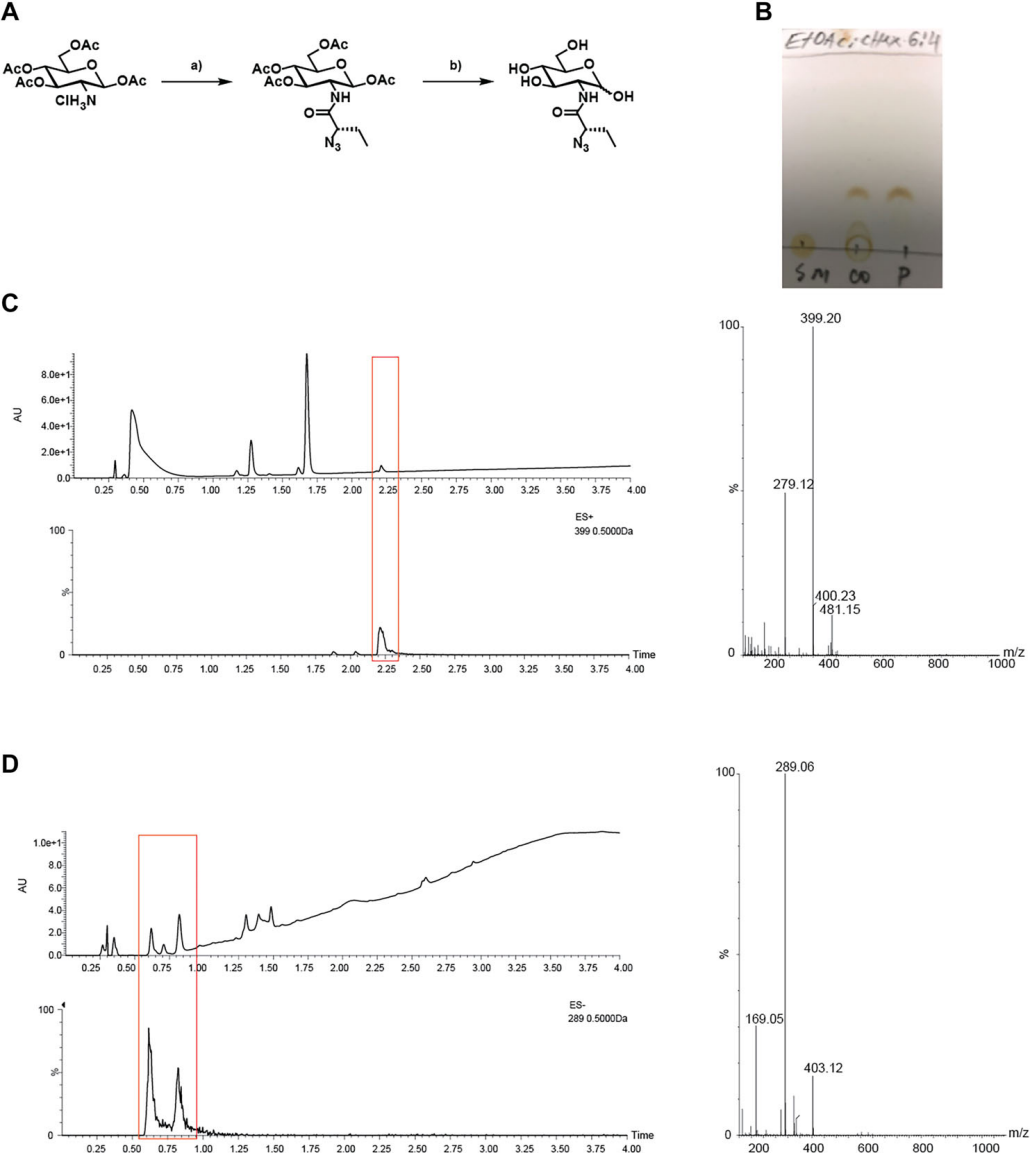
Example of reaction monitoring for the synthesis of GlcNEtAz(*S*). (**A**) Reaction scheme: a) amide coupling: (*S*)‐2‐azidobutyric acid; COMU, DIPEA, and DMF, room temperature (RT), 3–16 h, 51 %, *β‐*anomer only; b) deprotection: 1% NaOMe in MeOH, RT, 2 h, 44%, mixture of *α:β* in 1:0.3 ratio. (**B**) TLC after reaction: a) solvent mixture: cHex:EtOAc in 4:6 ratio; spots developed with “sugar stain”; (SM) starting material, (CO) co‐spot, and (P) product; reaction completion was confirmed by lack of starting material in the product spot. (**C**) UPLC‐MS spectra of the crude reaction mixture: a) UV trace (top) and extracted ion of the expected product *m/z* (bottom); the product peak is indicated with a red rectangle; on the right is the MS spectrum of the product peak (ESI); calcd. for C_16_H_23_N_4_O_8_^+^ (oxocarbenium ion) 399.15 and found 399.20 *m/z*. (**D**) UPLC‐MS spectra of the crude reaction mixture: UV trace (top) and extracted ion of the expected *m/z* (bottom); the product peak is indicated with a red rectangle; on the right is the MS spectrum of the product; peak MS (ESI) calcd. for C_10_H_18_N_4_O_6_ [M‐H^+^]^−^ 289.12 and found 289.06 *m/z*.

An example of TLC used for monitoring a reaction is shown in Figure 4B.

After heating, spots containing sugar become dark yellow or brown. The product spot should move at a higher R_f_ in relation to the SM. The reaction is considered complete when no SM is seen in the reaction mixture spot.

#### Reaction monitoring by UPLC‐MS or LC‐MS

Reaction monitoring by LC‐MS is optional. Take 10 µl of the reaction mixture and dilute 10‐fold with methanol and water 1:1 (v/v). Run the mixture on an LC‐MS instrument equipped with a C18 column and check the reaction progress by monitoring the diagnostic ions of the SM and product.

In our work, we use a UPLC‐MS instrument equipped with a C18 column. The standard analytical method used with this system is as follows: 1–9 µl injection volume, gradient of 3% to 95% buffer B over 2 min at a flow rate of 0.5 ml/min and column temperature at 50°C; buffer A: 0.1 % formic acid (v/v) and buffer B: 0.1% formic acid (v/v) in acetonitrile.

The GlcNAc/GalNAc analogues will be less hydrophilic than the SM and should elute later on a reverse‐phase column, albeit with little difference, as both the product and SM are poorly retained on this stationary phase. Longer run times can improve separation. Due to the poor retention and low UV absorbance of both the SM and the product, monitoring the reaction using MS signals is required. The optimal condition is MS in negative mode, but positive mode is also sufficient.

7 On completion of the reaction, remove the solvent using a rotary evaporator.

#### Purification using an automated flash chromatography system

8 Re‐dissolve the dry residue in methanol and dry load. Prepare the dry load by adding the required amount (as much as can be added to the prepacked cartridge) of silica or granulated sorbent, such as Isolute, to the dissolved residue and evaporate the solvent with the rotary evaporator until fully dry.
  Due to its hydrophilicity, the product is soluble only in polar solvents; hence, dry loading is recommended.
9 Load the dry silica or Isolute granules coated with the sample onto the solid phase extraction cartridge.
10 Connect the loaded column to the flash chromatography purification system and elute the product with a linear gradient of 0% to 30% buffer B over 10 column volumes. Buffer A: dichloromethane and Buffer B: methanol.
  Alternatively, a manually packed silica column can be used. Weigh out the amount of silica needed to pack a chromatography column with a filter and a PTFE stopcock. In a beaker, prepare a slurry with the silica gel in dichloromethane. Secure the column on a retort stand with a clamp and place an empty beaker underneath. Transfer the slurry into the column in one motion while keeping the stopcock closed. Leave the silica to settle and open the stopcock. Allow the solvent to exit the column while ensuring the silica gel bed does not run dry. Add more of the slurry and repeat the process until 2/3 of the column is packed. Applying a light positive pressure at the top of the column will speed up the process and ensure the column is tightly packed and free of air pockets. Add 1–2 medium‐sized spatulas of sand on top of the packed column and elute 20–50 ml of dichloromethane until only a thin layer (1–2 mm) of solvent is left above the sand. Add the dry loaded sample on the top and add 1–2 spatulas of sand over it. For a manually packed column, use as little silica/sorbent as possible for the dry load. This is the amount that will allow you to dry and scrape the entire contents of the flask. Wash with dichloromethane (50–100 ml) and elute the product with 30% methanol in dichloromethane. Collect the wash and elution volumes in separate 5‐ml fractions.
  Depending on the analogue, different elution gradients may be required. To optimize the gradient, refer to the R_f_ value of the product on the TLC plate.
  If an automated flash chromatography system is used, choose the option to collect all fractions to ensure no product is lost.
11 Identify the product containing fractions using TLC, as described in step 6.
12 Combine the fractions containing the product in a preweighed 100‐ or 250‐ml round‐bottom flask and evaporate the solvents with a rotary evaporator. Connect to a high vacuum to obtain the dry product, often as a white powder or foam.
13 Dissolve 5–10 mg of the product in methanol‐d4 and characterize using NMR.
  Apart from ^1^H and ^13^C NMR, 2D experiments such as COSY and HSQC are highly beneficial for characterizing carbohydrates.
  In most cases, the product still contains a considerable amount of triethylamine and traces of HOBT. The amount and presence of impurities will depend on the nature of the bioorthogonal tag, particularly its hydrophobicity. A second reverse‐phase purification is needed for most analogues. Analogues with larger alkyl chains will be more hydrophobic and can be separated from impurities by C18 solid‐phase extraction (see below). Smaller modifications are likely to be more hydrophilic and co‐elute with triethylamine. When the retention time of the product is very similar to the retention time of the impurity, HPLC purification is recommended. If HPLC also does not yield a pure product, refer to Alternative Protocol 1.
14 Take a small amount of the silica‐purified product (app. 1 mg), dissolve in methanol (app. 1 ml), and run on the LC‐MS system equipped with a C18 column.
  The chromatogram will show how close the retention times of the product and triethylamine are and indicate the next purification step. Triethylamine is not UV‐active, but it has a strong ion signal in positive mode, [M+H]^+^: 102 m/z. GlcNAc and GalNAc analogues will have a very weak UV signal at 210 nm due to the amide bond, but they show a strong ion signal in negative mode, [M‐H]^−^. When the difference in retention times between the product and triethylamine is too small, HPLC is recommended; otherwise, a C18 solid‐phase extraction step (see below) should yield a pure product.
15 Further purify the semipure product using solid phase extraction or HPLC.

#### Solid phase extraction using C18

a. Secure the solid‐phase cartridge on a retort stand with a clamp.
b. Equilibrate the cartridge by passing through 50 ml of acetonitrile, followed by 50 ml of deionized or HPLC grade water. Collect the flow‐through and discard appropriately.

c. Place a rack with test tubes under the column to be able to collect the eluants.
d. Dissolve the semipure product in a minimal amount of water (0.5–2 ml) and load onto the cartridge.
e. Wash with 20 ml deionized water. Allow the solvent to pass through the cartridge by gravity and collect the flow through in separate tubes as 2–5 ml fractions.
f. Elute the product with 20 to 50% (v/v) acetonitrile in deionized water (20 ml), while continuing to collect the flow‐through in 2–5 ml fractions.
  The percentage of acetonitrile will be determined by the elution time/% acetonitrile of the product in step 14. For example, if the product is eluting at ∼30% acetonitrile during analytical LC‐MS, use 30% (v/v) acetonitrile to elute it from the cartridge.
g. Identify the fractions containing the product by spotting all fractions, including the load and water wash, on a TLC plate.
h. Run each of the fractions identified to contain the product on the C18 column.
i. Combine all fractions that contain pure product and dry by lyophilization.

#### HPLC purification

a. Set up the process with the appropriate gradient and settings. For example, if the product elutes at ∼10% (v/v) acetonitrile during analytical LC‐MS, then the optimal gradient will be 0–15% (v/v) buffer B over 10 min (or CV).
  The exact duration of the gradient will also depend on the column, system, and flow rate used.
b. Dissolve the semipure product in a minimal amount of water (0.2–0.5 ml), inject, and run the HPLC process.
  Depending on the loading capacity of the column, several injections may be needed to purify the bulk semipure product.
c. Identify the pure fractions by reverse‐phase LC‐MS.
  We are using a mass‐directed autopurification system (MDAP) equipped with a C18 column. This allows for setting up collection triggered by mass, and direct assessment of the fraction purities is carried out. In this case, no further LC‐MS analysis of individual fractions is required. The typical flow rate is 25 ml/min, and the buffers are the same as those used for the analytical LC‐MS.
d. Combine all fractions that contain pure product and dry by lyophilization.

16 Weigh out the dry product and calculate the yield.
17 Dissolve 5–10 mg of the product in methanol‐d4 and characterize using NMR and LC‐MS.
  Typically, the product is a mixture containing α and β anomers in different ratios, with yields between 20% and 50%.
  Representative characterization data for analogue GlcNButAz obtained as an anomeric mixture in an α:β ratio of 6:5 and 48 % yield are given below.

^1^H NMR (400 MHz, D_2_O) δ 5.21 (d, *J* = 3.5 Hz, 0.55H), 4.72 (d, *J* = 8.4 Hz, 0.45H), 3.93–3.82 (m, 2H), 3.82 –3.67 (m, 2H), 3.58–3.43 (m, 2H), 3.38 (td, *J* = 6.8, 1.6 Hz, 2H), 2.40 (td, *J* = 7.3, 5.3 Hz, 2H), 1.90 (m, *J* = 7.0 Hz, 2H); ^13^C NMR (100 MHz, D_2_O) δ 176.4, 176.2, 94.9, 90.8, 75.9, 73.8, 71.5, 70.6, 70.1, 69.9, 60.7, 60.6, 56.5, 54.0, 50.4, 50.2, 33.1, 32.8, 24.5, 24.5. The calculated MS (ESI) for C_10_H_18_N_4_O_6_ (M‐H^+^) was 289.12, and the found value was 289.05 *m/z*.

^1^H and ^13^C NMR, COSY, and HSQC spectra are shown in Supplementary Figure 1.

18 Store the GlcNAc/ GalNAc analogues in a lyophilized form at −20 °C.
  Precise stability testing has not been carried out; however, they are stable for up to 5 years when stored at −20 °C.

## Alternate Protocol 1 CHEMICAL SYNTHESIS OF BIOORTHOGONALLY TAGGED ACYLAMIDE ANALOGUES OF D‐GlcNAc AND D‐GalNAc FROM A PROTECTED GlcNH_2_ or GalNH_2_ PRECURSOR

Some branched or small bioorthogonal tags may be difficult to couple to unprotected GlcNH_2_ or GalNH_2_ following Basic Protocol 1. In addition, the purification of the product from the reaction mixture is more challenging, often leading to further loss of yield. This alternative protocol offers a method that overcomes both challenges by reacting the acid with a tetra‐acetylated sugar precursor (Choi et al., 2019; Dang et al., 2014). The resulting protected sugar analogue is easily extracted from the reaction mixture with an organic solvent and purified in good yields. Subsequent deprotection of the acetyl groups with sodium methoxide and normal phase chromatography gives the final pure GlcNAc or GalNAc analogues in moderate yields.

*CAUTION*: All reactions must be carried out in a suitable, well‐ventilated fume hood. Lab coat, protective gloves, and safety goggles must be worn when handling reagents and reaction mixtures.

*CAUTION*: Always use a secondary container in which to place the reaction vessel. The secondary container should be able to hold the entire contents of the primary container plus an extra 10% in volume.

### Additional Materials

1,3,4,6‐tetraacetyl glucosamine hydrochloride (Ac_4_GlcNH_2_.HCl) (prepared according to Biswas et al., 2017)
1,3,4,6‐tetraacetyl galactosamine hydrochloride (Ac_4_GalNH_2_.HCl) (prepared according to Biswas et al., 2017)
Carboxylic acid containing a bioorthogonal tag
*N,N*‐dimethylformamide, anhydrous, 99.8% (DMF; Sigma‐Aldrich, cat. no. 227056, CAS No: 68‐12‐2)
*N*,*N*‐Diisopropylethylamine (DIPEA; Sigma‐Aldrich, cat. no.: D125806, CAS No: 7087‐68‐5)
1‐cyano‐2‐ethoxy‐2‐oxoethylidenaminooxy)dimethylamino‐morpholino‐carbenium hexafluorophosphate (COMU; Sigma‐Aldrich, cat. no. 712191, CAS No: 1075198‐30‐9)
Cyclohexane, HPLC grade (Sigma‐Aldrich, cat. no. 650455, CAS No: 110‐82‐7)
Ethyl acetate, HPLC grade (Sigma‐Aldrich, cat. no. 34858, CAS No: 141‐78‐6)
Chloroform‐d (Sigma‐Aldrich, cat. no. 151823, CAS No: 865‐49‐6)
Sodium methoxide solution, 25% in methanol (Sigma‐Aldrich, cat. no. 156256, CAS No: 124‐41‐4)
DOWEX 50W X8 Ion Exchange Resin, hydrogen form, strongly acidic, 50–100 mesh (Sigma‐Aldrich, CAS no. 69011‐20‐7)

UPLC‐MS system (Acquity H‐Class PLUS qDA UPLC‐MS, Waters, UK) equipped with a C18 column (ACQUITY UPLC BEH C18, 130 Å, 1.7 µm, 2.1 × 50 mm)
Sfär Silica D Duo 25 g cartridge (Biotage)
Flash chromatography purification system (Isolera One flash chromatography system or equivalent)

*CAUTION*: DMF, cyclohexane, ethyl acetate, 3‐methoxyphenol, ethanol, and sodium methoxide are flammable, toxic, and irritant chemicals. Sulfuric acid is corrosive. Wear a lab coat, protective gloves, and safety goggles when handling them. These reagents should be handled in a fume hood with care.

#### Amide coupling

1 Weigh out Ac_4_GlcNH_2_.HCl or Ac_4_GalNH_2_.HCl (180 mg, 0.47 mmol) and transfer into a flame‐dried, 25‐ml round‐bottom flask equipped with a magnetic stirrer bar. Close the flask with a rubber stopper and connect to a Schlenk line. Open the N_2_ line and let the gas flow into the flask. Transfer anhydrous DMF (5 ml) into the flask with a syringe and needle and start stirring by positioning the flask above a magnetic plate stirrer.
2 Add the carboxylic acid containing a bioorthogonal tag (0.52 mmol, 1.1 eq.) as a solid or with a syringe and needle. Cool the solution to 0 °C by placing the flask in an ice‐filled container while continuing to stir and keeping it under an inert atmosphere.
3 Measure DIPEA (245 µl, 1.41 mmol, 3 eq.) with a syringe and needle and add it to the mixture by inserting the needle into the rubber septum and dispensing the contents of the syringe.
4 Remove the septum and quickly add COMU (201 mg, 0.47 mmol, 1 eq.) to the reaction mixture and warm to room temperature by removing the ice bath.
  At this step, the reaction mixture will become dark orange to brown.
5 Leave the reaction mixture to stir for 3 to 16 h at room temperature and under N_2_.
  Start monitoring the reaction from 3 h onward using TLC or/and LC‐MS.
  If the SM is still present, add more of the acid (1.1 eq.) and COMU (1 eq.) and leave to stir until all SM is consumed.
6 Monitor the reaction using TLC or/and LC‐MS.
  Reaction monitoring is shown in Figure 4.

#### Reaction monitoring by TLC

Solvent mixture: cyclohexane: ethyl acetate 1:1 (v/v)

The exact ratio of the solvent mixture may vary depending on the bioorthogonal tag.

“Sugar stain”: 0.1% (v/v) 3‐methoxyphenol and 2.5% (v/v) sulfuric acid in ethanol (see recipe)

With a pencil, draw a horizontal line near the bottom of the TLC plate. Spot the following separate samples along this line: (A) starting material (SM), (B) co‐spot (with both SM and reaction mixture), and (C) reaction mixture. Develop the plate by placing it in a TLC chamber with 1:1 (v/v) cyclohexane: ethyl acetate and run it until the solvent line is close to the top of the TLC plate.

The solvent line in the TLC chamber should be below the spots.

Leave the TLC to dry for a few minutes in a fume hood, dip it in the sugar stain, and heat it with a heating gun until dark yellow to brown spots appear.

An example of TLC used for monitoring this reaction is shown in Figure 4B.

After heating, spots containing sugar become dark yellow or brown. The product spot should move up the plate in relation to the SM.

#### Reaction monitoring using UPLC‐MS or LC‐MS (optional)

Take a small sample of the reaction mixture and further dilute 10‐fold with 1:1 (v/v) methanol and water. Run LC‐MS equipped with a C18 column and check the completion by comparing the ions of the SM and the product.

We used UPLC‐MS. The standard analytical method used with this system is as follows: 1–9 µl injection volume, gradient of 3% to 95% buffer B over 2 min at a flow rate of 0.5 ml/min and column temperature at 50° C; buffer A: 0.1 % formic acid (v/v) and buffer B: 0.1% formic acid (v/v) in acetonitrile.

The GlcNAc/GalNAc analogues will be more hydrophobic than the SM and should elute later. The major product ion seen in positive MS mode is the oxonium ion [M‐CH_3_COO^−^] ^+^.

7 On completion of the reaction, evaporate the solvent using a rotary evaporator.

#### Purification

8 Re‐dissolve the dry residue in a minimal amount (0.5–1 ml) of chloroform and load onto the Sfär Silica or an alternative silica‐loaded cartridge.
  Typically, the residue is well dissolved in 0.5–1 ml chloroform and dry loading is not necessary.
9 Connect the loaded column to the flash chromatography purification system and elute the product with a linear gradient of 0% to 40% Buffer B over 10 CV. Buffer A: cyclohexane and Buffer B: ethyl acetate.
  Alternatively, a manually packed silica column could also be used. Refer to Basic Protocol 1, step 10 for more information on how to pack a column.
  Depending on the analogue, different elution gradients may be required. To optimize the gradient, refer to the R_f_ value of the product on the TLC plate.
  If an automated flash chromatography system is used, choose the option to collect all fractions to ensure no product is lost due to its low UV absorbance.
10 Identify the product containing fractions using TLC as described in Basic Protocol 1, step 6.
11 Combine the fractions containing the product in a tared 100‐ or 250‐ml round‐bottom flask and evaporate the solvents with a rotary evaporator.
12 Attach the flask to a high vacuum line for final drying.
  The final product is a yellow oil or white powder.
13 Weigh out the dry product and calculate the yield.
  Typically, the product is only the ‐β anomer with yields between 50% and 70%.
14 Dissolve 5–10 mg of the product in chloroform‐d and characterize it using NMR. Run ^1^H, ^13^C, COSY, and HSQC experiments.
  Representative characterization data for analogue Ac_4_GlcNEtAz(S) obtained as a yellow oil (51%, β‐anomer only) are given below.

^1^H NMR (400 MHz, CDCl3) δ 6.42 (d, *J* = 9.4 Hz, 1H), 5.74 (d, *J* = 8.7 Hz, 1H), 5.23 (dd, *J* = 10.5, 9.3 Hz, 1H), 5.14 (t, *J* = 9.5 Hz, 1H), 4.31–4.19 (m, 2H), 4.13 (dd, *J* = 12.5, 2.3 Hz, 1H), 3.91 (dd, *J* = 6.6, 4.9 Hz, 1H), 3.82 (m, *J* = 9.8, 4.6, 2.3 Hz, 1H), 2.09 (d, *J* = 1.9 Hz, 6H), 2.04 (d, *J* = 2.8 Hz, 6H), 1.94–1.77 (m, 2H), 0.95 (t, *J* = 7.4 Hz, 3H); ^13^C NMR (100 MHz, CDCl3) δ 171.0, 170.8, 169.7, 169.4, 169.3, 92.4, 73.1, 72.2, 67.8, 65.6, 61.7, 53.2, 25.4, 20.9, 20.9, 20.7, 20.7, 9.5. The calculated MS (ESI) for C_16_H_23_N_4_O_8_^+^ (oxocarbenium ion) is 399.15 and the found value is 399.08 *m/z*.

^1^H, ^13^C NMR, COSY, and HSQC spectra are shown in Supplementary Figure 2A.

15 Store the Ac_4_GlcNAc/GalNAc analogues at 4°C for shorter periods or at −20°C for longer periods.
  Precise stability testing has not been carried out; however, they are stable for up to 5 years when stored at −20°C.

#### Deprotection of tetra‐acetylated sugar analogues

16 Transfer 50 mg of the tetra‐acetylated GlcNAc or GalNAc analogue from step 15 to a 25‐ml round bottom flask equipped with a magnetic stirrer.
17 Prepare 2 ml 1% (w/v) sodium methoxide in methanol and add to the flask.
18 Leave to stir at room temperature for 2–5 h.
19 Monitor the reaction with TLC or LC‐MS, as described in Basic Protocol 1, step 6.
20 Transfer ∼5 g of the hydrogen form resin into a filtered syringe and wash several times with deionized water.
  Initially, the water that comes off the resin is a dark orange color. Wash until the water passing through becomes clear. Use either gravity or pressure to wash the resin.
21 On completion of the reaction, add the washed resin and leave to stir for 10 min.
22 Filter and rinse the resin with 2–5 ml methanol, followed by 2–5 ml water; collect both the filtrate and wash in a round‐bottom flask.
23 Evaporate the solvent using a rotary evaporator and high vacuum and/or lyophilization.

#### Purification

24 Redissolve the dry residue in methanol, dry load, elute, and collect the pure fractions, as described in Basic Protocol 1, steps 8 to 11.
25 Combine the fractions containing the product in a tared 100‐ or 250‐ml round‐bottom flask and evaporate the solvents with a rotary evaporator.
26 Attach the flask to a high vacuum line for final drying.
  The final product is a white powder.
27 Weigh out the dry product and calculate yield.
  Typically, the product is a mixture of α and β in different ratios, with yields between 40% and 60%.
28 Dissolve 5–10 mg of the product in methanol‐d4 and characterize using NMR. Run ^1^H, ^13^C, COSY, and HSQC experiments.
  Representative characterization data for analogue GlcNEtAz(S) obtained as a white powder (44 %, 7:3 α:β) are given below.

^1^H NMR (400 MHz, CD3OD) δ 5.12 (d, *J* = 3.5 Hz, 0.7H), 4.65 (d, *J* = 8.3 Hz, 0.3H), 4.06–3.41 (m, 6H), 3.40–3.34 (m, 28, 1H), 2.01–1.71 (m, 2H), 1.02 (td, *J* = 7.4, 5.2 Hz, 3H); ^13^C NMR (100 MHz, CD3OD) δ 172.6, 96.8, 92.5, 78.0, 75.8, 73.2, 72.6, 72.5, 66.0, 65.5, 62.8, 58.8, 55.9, 26.3, 26.1, 10.2, 10.2. The calculated MS (ESI) for C10H18N4O6 (M‐H+) is 289.12 and the found value is 289.1 *m/z*.

^1^H and ^13^C NMR, COSY, and HSQC spectra are shown in Supplementary Figure 2B.

29 Store the dried product at −20°C.

## Basic Protocol 2 CONVERSION OF D‐GlcNAc OR D‐GalNAc ANALOGUES TO UDP‐SUGARS ON ANALYTICAL (REACTION SCOUTING) AND PREPARATIVE SCALES BY CHEMOENZYMATIC SYNTHESIS AND PURIFICATION

Prior to a preparative‐scale enzymatic synthesis of UDP‐GlcNAc/GalNAc analogues, an analytical‐scale screening is carried out. Each analogue is tested against a reaction mixture containing NahK, PmPpA, and each of the two AGX1 variants to establish the optimal conditions for the large‐scale enzymatic reaction. The bacterial kinase NahK (*B. Longum*, Chemily Glycoscience, Peachtree Corners, USA) is widely promiscuous, quantitatively converting all analogues we have tested to their respective GlcNAc‐1‐P and GalNAc‐1‐P analogues. The pyrophosphorylases used are AGX1 WT and the variant F383A. Inorganic phosphatase PmPpA (*P. Multocida*, Chemily Glycoscience, Peachtree Corners, USA) is added to the enzymatic reaction to drive the forward reaction by hydrolyzing the pyrophosphate byproduct of the reaction. ATP and UTP are used as phosphate and uridyl phosphate donors, respectively. Reactions are left to proceed for 16 h, and analogue turnover is determined using LC‐MS. In our hands, different analogues were tolerated to different degrees by the WT and F383A enzymes, emphasizing the benefit of using AGX1 F383A and analytical‐scale screening prior to preparative‐scale synthesis. The analysis should show which AGX1 variant results in the highest turnover for each of the analogues used. A preparative‐scale enzymatic reaction should then be set up with the optimal AGX1 variant and the pure product isolated from the reaction mixture using a combination of enzymatic digestion and chromatography.

### Materials

GlcNAc analogues (synthetized in Basic Protocol 1 or Alternate Protocol 1)
Adenosine 5′‐triphosphate disodium salt hydrate (ATP; Sigma‐Aldrich, cat. no. A2383, CAS No: 34369‐07‐8)
Uridine 5′‐triphosphate trisodium salt hydrate (UTP; Sigma‐Aldrich, cat. no. U6625, CAS No: 19817‐92‐6)
N‐acetylhexosamine 1‐kinase (NahK), *E. coli* recombinant N‐acetylhexosamine 1‐kinase from *Bifidobacterium longum* (Chemily Glycoscience, cat. no. EN01011, E.C. no. 2.7.1.162)
Inorganic pyrophosphatase (PmPpA), *E. coli* recombinant from *P. Multocida* (Chemily Glycoscience, cat. no. EN01018, E.C.No.: 3.6.1.1)
AGX1 WT and AGX1 F383A, previously expressed and purified (Cioce et al., 2022)
  Plasmids are available from the corresponding authors upon request.
Reaction buffer, 2× (see recipe)
Acetonitrile, Optima LC‐MS grade (Fisher Scientific, cat. no. 10001334, CAS No: 75‐05‐8)
Uridine 5′‐diphospho‐N‐acetylgalactosamine disodium salt (UDP‐GalNAc) (Sigma‐Aldrich, cat. no. U5252, CAS No: 108320‐87‐2)
Uridine 5′‐diphospho‐N‐acetylglucosamine sodium salt (UDP‐GlcNAc) (Sigma‐Aldrich, cat. no. U4375, CAS No: 91183‐98‐1)
Bovine Serum Albumin (BSA; Sigma‐Aldrich, cat. no. A2153‐10g, CAS no: 9048‐46‐8)
Phosphate Buffered Saline (PBS; Sigma‐Aldrich, cat. no. P3813)
Calf intestinal alkaline phosphatase (CIAP; Invitrogen, cat. no. 18009‐019)
Ethanol (Sigma‐Aldrich, cat. no. 493546, CAS no: 64‐17‐5)
Acetonitrile, HPLC grade (Sigma‐Aldrich, cat. no. 34851, CAS no: 75‐05‐8)
Ammonium formate (Sigma‐Aldrich, cat. no. 70221, CAS no: 540‐69‐2)
Dowex 50W X8 Ion Exchange Resin, sodium form, strongly acidic, 100–200 mesh (Sigma‐Aldrich, CAS no. 69011‐22‐9)

Incubator (Infors HT or equivalent)
Microcentrifuge with PCR tubes adaptors (Fresco 17, Thermo Scientific or equivalent)
Large centrifuge (Eppendorf 5920 R G or equivalent)
50‐ml centrifuge tubes
Liquid chromatography‐mass spectrometry (LC‐MS) or Ultra‐performance liquid chromatography‐mass spectrometry (UPLC‐MS) [Acquity H‐Class PLUS qDA UPLC‐MS, Waters, equipped with ACQUITY UPLC Glycan BEH Amide column (130 Å, 1.7 µm, 2.1 × 100 mm) or similar]
Rotary evaporator (Büchi 300 or equivalent)
Solid phase extraction cartridge (Sep‐Pak C18 20 cc Vac Cartridge, 5 g, Waters, or equivalent).
High‐performance liquid chromatography purification system (Agilent 1260 Infinity II MDAP system, Agilent Technologies) equipped with a XBridge BEH Amide OBD Prep column (130 Å, 5 µm, 10 mm × 100 mm column or equivalent)
Nuclear magnetic resonance (NMR) instrument (Bruker Avance‐400 MHz or above or equivalent)

*CAUTION*: Acetonitrile is an irritant and toxic and should be handled with care.

#### Conversion of D‐GlcNAc or D‐GalNAc analogues to UDP‐sugars on an analytical scale (reaction scouting)

The experiment below is an example of an analytical reaction set up for a GlcNAc analogue.

**Figure 5:**
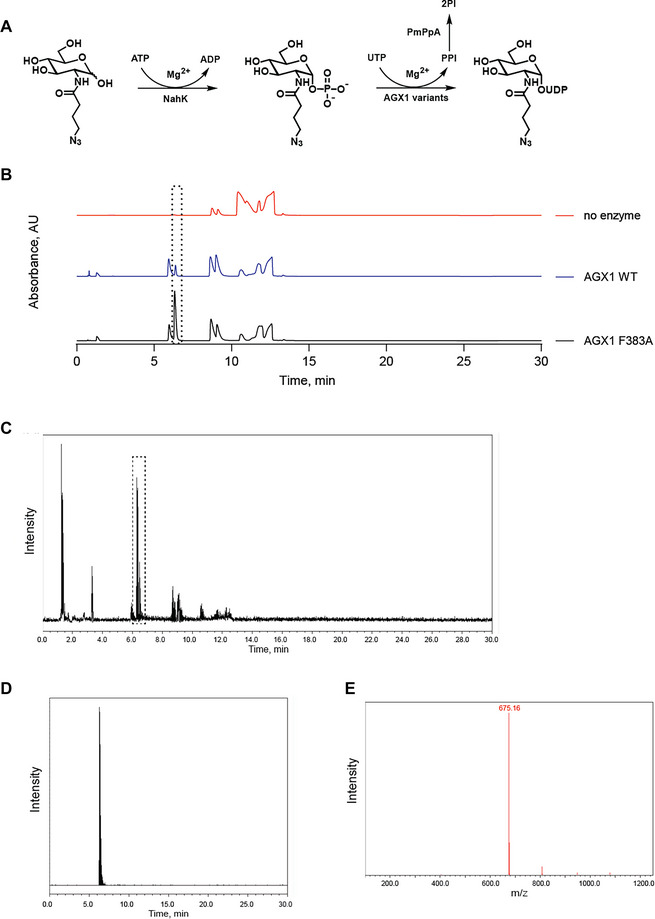
(**A**) Reaction scheme. (**B**) Overlaid chromatograms of analytical scale reactions with GlcNButAz and no enzyme (top), AGX1 WT (middle), and AGX1 F383A (bottom); UV signal at 260 nm; product peaks at 6.3 min are indicated with a dotted rectangle; later peaks correspond to the excess ATP and UTP and the reaction byproduct ADP. (**C**) Total ion chromatogram (TIC) of the reaction with AGX1 F383A. Product peak indicated with a dotted rectangle. (**D**) Extracted 675.11 ion spectrum of expected product mass in negative mode. (**E**)The product ion corresponding to the TIC and extracted peak at 6.3 min; MS (ESI) calcd. for C_19_H_29_N_6_O_17_P_2_ [M‐H^+^]^−^ 675.11 and found 675.16 *m/z*.

1 Prepare 50 mM solutions of GlcNAc analogue, ATP, and UTP in MilliQ water.
  Each analytical reaction will require very small amounts of the solutions. Prepare 50 mM stock solutions (0.5–1 ml), store at −20°C, and defrost shortly before setting up the reactions. These solutions are stable and can withstand multiple freeze–thaw cycles.
2 Take out NahK, recombinant AGX1 variants, and PmPpA from the −80 °C freezer and allow them to thaw on ice.
  Prepare stock solutions of enzymes in 20 % glycerol in advance, aliquot into small (for example, 5 or 10 µl) volumes in microcentrifuge tubes, and store at −80 °C. NahK (0.0025U/*µl*), *PmPpA*(0.005*U*/*µl*) and AGX1 (5 µM). This is to avoid repeated freezing and thawing, which can alter enzyme activity. Take aliquots out of −80 °C and only use each aliquot once. Avoid prolonged exposure at room temperature.
3 Prepare the 2× Reaction buffer as described in the recipe.
  Tris·HCl and MgCl_2_ stock solutions can be stored at room temperature for up to several months, while BSA can be stored at 4°C for a month. Prepare the 2× Reaction buffer directly before the reaction is set up and discard any leftovers at the end of the experiment.
4 Take 3 PCR tubes and add 10 µl of the 2× Reaction buffer to each.
5 Add 10 µl of Milli‐Q water to the first tube. This tube will contain all components of the reaction mixture except for the enzymes and will be used as a blank to normalize the signal by deducting any potential background noise.
6 Add 4 µl of milliQ water to tubes 2 and 3.
7 To tubes 2 and 3, add 2 µl NahK (stock solution 0.0025 U/µl) and 2 µl PmPpA (stock solution 0.005 U/µl).
8 Add 2 µl from AGX1 WT (stock solution 5 µM) to tube 2 and 2 µl from AGX1 F383A (stock solution 5 µM) to tube 3.
  The total reaction mixture volume is 20 µl. The final concentration of the mixture is as follows: 2.5 mM GlcNAc analogue, 5 mM ATP, 5 mM UTP, 100 mM Tris·HCl, 5 mM MgCl_2_, 1 mg/ml BSA, 0.0025 U NahK, 0.005 U PmPpA, and 500 nM AGX1 WT and variant.
9 Gently mix each reaction mixture (tubes 1–3) by pipetting and incubate the tubes at 37°C for 16 h.
10 After 16 h, add 20 µl of ice‐cold acetonitrile to each tube and place all reactions on ice for 30 min.
11 Centrifuge the tubes for 30 min at 4°C and 16,200 × *g*.
  The addition of cold acetonitrile and centrifugation will ensure that the protein is precipitated, forming small pellets at the bottom of the vial. The addition of acetonitrile will also improve the chromatographic separation.
12 Take out the tubes from the centrifuge and transfer the supernatant (30 µl) into the LC‐MS insert vials.
  At this scale, the pellet may not be visible, but it is important to remove the proteins prior to monitoring the reactions by LC‐MS, especially when injecting into a HILIC column. Take out the supernatant without disturbing the vial, ensuring the pipette tip is not touching the bottom of the vial.
13 Run the samples on the LC‐MS instrument equipped with a Glycan column or equivalent. Run each sample twice to obtain an average value of the two technical replicates for each sample.
  We used UPLC‐MS. The standard analytical method used with this system is as follows: 9 µl injection volume, gradient of 90%–55 % buffer B over 17 min at a flow rate of 0.35 ml/min, and column temperature at 50°C; buffer A: 10 mM ammonium formate pH 4.5 and buffer B: 10 mM ammonium formate in 90:10 v/v acetonitrile: water. The sample manager (sample tray) is kept at 10°C to prevent evaporation of the acetonitrile in the samples and ensure sample consistency regardless of the order of analysis.
14 Analyze the LC‐MS data by integrating the peak area of the product at 260 nm. MS in negative mode will confirm the product as [M‐H^+^]^−^ ions. Use the blank sample to check if a potential background signal is present in the area of the product peak. If a background signal is present, normalize the data by deducting the background integral value from each of the recorded values.
  An example of the reaction analysis for one GlcNAc analogue is shown in Figure 5.

#### Standard curve sample preparation

**Figure 6:**
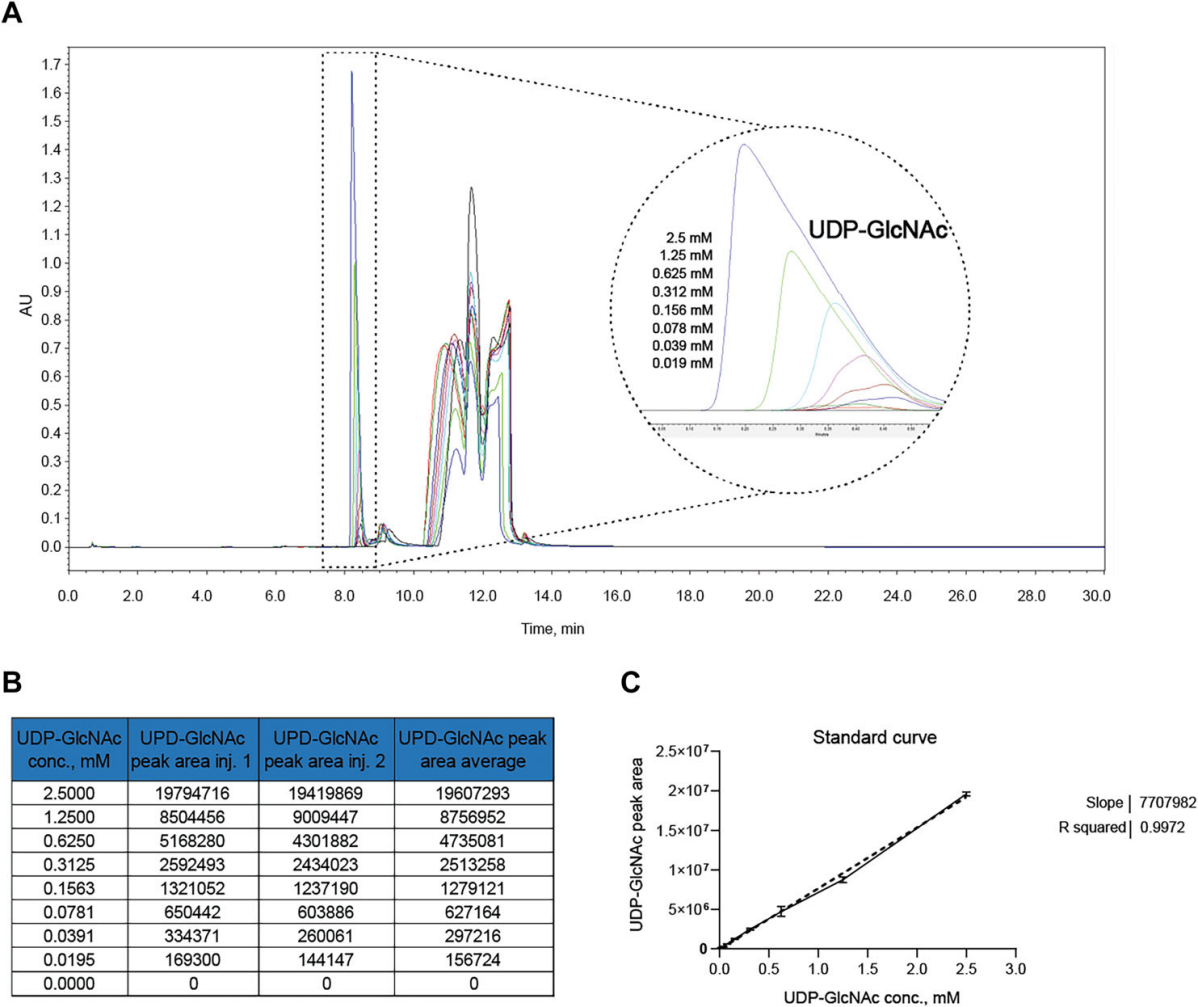
(**A**) UV spectra of standard curve samples at 260 nm prepared by serial dilutions of UDP‐GlcNAc with concentrations of 2.5 mM, 1.25 mM, 0.625 mM, 0.3125 mM, 0.156 mM, 0.078 mM, 0.039 mM, and 0.0195 mM. UDP‐GlcNAc peaks are indicated with a black dotted rectangle. (**B**) Values for the peak area of UDP‐GlcNAc for each concentration. Values were obtained using EmPower software (Waters, USA). (**C**) Standard curve of UDP‐GlcNAc with slope and R‐squared values calculated using GraphPad Prism 10 (Dotmatics, USA). The dotted line represents the best fit of the linear regression curve.

15 Prepare serial dilutions of UDP‐GlcNAc with concentrations of 5 mM, 2.5 mM, 1.25 mM, 0.625 mM, 0.3125 mM, 0.156 mM, 0.078 mM, and 0.039 mM in deionized water with a volume of 10 µl each in microcentrifuge tubes and label them 1 to 8. Include tube 9 with 10 µl of Milli‐Q water. Tube 9 will be the blank sample containing all standard curve reagents except the product.
16 Add 10 µl of 2× Reaction buffer to tubes 1 to 9 and mix thoroughly.
  The total reaction mixture volume is 20 µl. The final concentration of the mixture is as follows: 2.5 mM GlcNAc, 5 mM ATP, 5 mM UTP, 100 mM Tris·HCl, 5 mM MgCl_2_, 1 mg/ml BSA, and UDP‐GlcNAc in concentrations from 2.5 to 0.0195 mM. This ensures that the standard curve sample components are close to the reaction mixture samples, ensuring coverage of a wide range of product concentrations.
17 Add 20 µl of acetonitrile to each tube and mix thoroughly.
18 Treat the standard curve samples as the reaction samples from steps 11 to 13. Run each sample twice to obtain an average value for each concentration.
19 Analyze the LC‐MS data by recording the integration of the peak area of UDP‐GlcNAc at 260 nm. Normalize data if needed, as described in step 14.
20 Construct the standard curve by plotting the average integrated peak area for each of the known UDP‐GlcNAc concentrations used. Record the slope of the curve.
  An example of the standard curve for UDP‐GlcNAc is shown in Figure 6.
  Note: The absolute values of the peak areas will depend on the instrument used.

21 Calculate the product concentration of samples 2 and 3 by dividing the values taken in step 14 by the slope of the standard curve from step 20. The percentage conversion is calculated with the following formula:

(Product concentration /2.5) ×100 = % turnover

The AGX1 variant that has resulted in the highest % turnover for this GlcNAc analogue will be used for its preparative‐scale enzymatic synthesis to the corresponding UDP‐GlcNAc analogue.

Most analogues in our hands were converted to a reasonable degree after 16 h. If necessary, longer times or more AGX1 variants can be added to aid the completion of the reaction.

#### Preparative‐scale synthesis and purification of UDP‐GlcNAc and UDP‐GalNAc analogues

The experiment below is an example of a preparative reaction set up for one GlcNAc analogue.

##### The same protocols for synthesis and purification can also be used for GalNAc analogues

22 Weigh out the GlcNAc analogue (0.03 mmol), ATP (0.06 mmol), and UTP (0.06 mmol).
23 Transfer the compounds into a 50‐ml centrifuge tube and dissolve in 3.4 ml Milli‐Q water.
  This will make up a final concentration of 2.5 mM for the analogue and 5 mM for the ATP and UTP in the total reaction mixture volume of 12 ml.
24 Transfer 1.2 ml Tris·HCl buffer stock solution (1 M, pH 8), 1.2 ml MgCl_2_ buffer stock solution (50 mM), and 1.2 ml BSA buffer stock solution (10 mg/ml in PBS) into the tube and mix well by inversion.
25 Add 2.980 ml of Milli‐Q water and mix well by gently inverting the tube several times.
26 Transfer 800 µl NahK stock solution (0.0025U/µl), 20 µl PmPpA stock solution (0.5U/µl), and 1.2 ml AGX1 variant stock solution (5 µM).
  Mix gently, as BSA will cause the solution to foam if shaken vigorously.
  The final volume of the reaction mixture is 12 ml.
  The AGX1 variant is the one determined to result in maximum conversion, as determined in step 21.
  The final concentration of the components in the reaction mixture is as follows: 2.5 mM GlcNAc analogue, 5 mM ATP, 5 mM UTP, 100 mM Tris·HCl, 5 mM MgCl_2,_ 1 mg/ml BSA, 2U NahK, 10 U PmPpA, and 500 nM AGX1.
27 Leave the reaction mixture in a shaking incubator at 37 °C and 220 × *g* for 16 h.
28 After 16 h, transfer 10 µl of the reaction mixture into a microcentrifuge tube, add 10 µl of cold acetonitrile, and place on ice for 30 min.
29 Treat the sample as in steps 11 to 13 and calculate the percentage conversion as in step 21, using the same standard curve.
  If the turnover has not reached the maximum observed in the analytical‐scale preparation, add more AGX1 variant and leave to incubate for a further 16 h. Incubate until the observed maximum turnover is reached or the reaction progress is stalled.
30 Once the reaction has stopped progressing, add CIAP (10 U) and leave the reaction mixture to incubate for a further 16 h at 37°C.
  The addition of CIAP will aid the purification of the final product. Calf intestinal alkaline phosphatase hydrolyzes the excess ATP and UTP, as well as ADP, the reaction byproducts. These hydrolyzed nucleotides (nucleosides), unlike their non‐hydrolyzed counterparts, will have different hydrophilicity and their retention time will be significantly different to that of the product, allowing for more straightforward purification.
31 Add 12 ml of ice‐cold ethanol to the reaction mixture and leave on ice for 30 min.
  The ethanol will denature protein, which will become a white precipitate that will be easily removed by centrifugation.
32 Centrifuge the tube for 30 min at 4 °C and 16,200 × *g*.
33 Transfer the supernatant to a 50‐ml round‐bottom flask and discard the pellet.
34 Remove the ethanol from the supernatant using a rotary evaporator.
35 Lyophilize the remaining aqueous solution.
36 Pre‐equilibrate a C18 solid phase extraction cartridge as described in Basic Protocol 1, step 15.
37 Dissolve the dried residue in a minimal amount of water and load onto the pre‐equilibrated C18 solid phase extraction cartridge.
38 Elute first with 20 ml water, followed by 20 ml 30% acetonitrile, collecting everything as 2–5 ml fractions.
  This step is recommended to remove salts from the reaction mixture residue and to allow higher loading for subsequent HPLC purification.
39 Identify all fractions containing the product by either TLC or/and LC‐MS, as described in Basic Protocol 1, step 6.
  Analyzing a small sample from each fraction by LC‐MS equipped with a HILIC column will show if further purification is needed.
  Typically, LC‐MS analysis shows fractions containing a mixture of the product and some nucleosides. In most cases, HPLC purification is required. A HILIC column is recommended as an effective method of separating a mixture of hydrophilic compounds.
40 Collect all fractions containing the product in a 20‐ml glass vial.
41 Take a small sample (10 µl) of the collected fractions in step 40 and transfer it into an LC‐MS insert vial. Add acetonitrile (10 µl), mix well by pipetting a few times, and run on the LC‐MS instrument equipped with the HILIC column. The buffers and gradient are detailed in step 13.
  Running a sample on the analytical column will indicate what gradient to create for the preparative‐scale purification. This step can be omitted if the fractions were already run on an analytical HILIC column.
42 Lyophilize the rest of the combined fractions.
43 Purify the dried residue using a preparative HPLC system, equipped with a HILIC column.
  a. Set up a process with appropriate gradient and settings. For example, if the product is eluting at 70% Buffer B during the analytical LC‐MS run in step 41, the optimal purification gradient will be 90%–60% Buffer B over 10 min (or 10 CV)
    The exact duration of the gradient will also depend on the column, flow rate, and system that is used. We used a MDAP system equipped with a prep column. Buffers used were A: 10 mM ammonium formate at pH 4.5; B: 10 mM ammonium formate in 90:10 (v/v) acetonitrile: water, at a flow rate of 19 ml/min.
  b. Identify the pure fractions, combine them in a 20‐ml glass vial, and lyophilize.
    Using MDAP allows for setting up fraction collection triggered by mass and/or UV. In this case, direct assessment of the purities is carried out, and no further LC‐MS analysis of individual fractions is required. Alternatively, fraction collection can be based on the UV signal. In this case, analyzing each fraction using LC‐MS is recommended to confirm the purity of each fraction.
44 Transfer ∼5 *g* of the sodium form resin into a filtered syringe and wash several times with methanol and deionized water.
  Initially, the methanol and water that come off the resin are a dark orange color. Wash until the water passing through becomes clear. Use either gravity or positive or negative pressure to wash the resin.
45 Dissolve the dried pure product in a minimal amount of water and pass through the pre‐washed Na^+^ resin, eluting with water.
46 Collect the fractions that contain the product and lyophilize.
47 Weigh out the dried product and calculate the yield.
48 Dissolve 5–10 mg of the product in deuterium oxide and characterize using NMR and UPLC‐MS.
  Typically, the product is a white powder with yields between 30% and 70%.
  Representative characterization data for the UDP‐GlcNPrAz3Me(S) analogue obtained as a white powder (65%) are given below.

^1^H NMR (400 MHz, D_2_O) δ 7.88 (d, *J* = 8.1 Hz, 1H), 5.96–5.84 (m, 2H), 5.44 (dd, *J* = 7.2, 3.3 Hz, 1H), 4.32 – 4.26 (m, 2H), 4.15 (dddt, *J* = 24.7, 14.9, 8.7, 3.0 Hz, 3H), 3.97–3.69 (m, 6H), 3.47 (dd, *J* = 10.1, 9.1 Hz, 1H), 2.54–2.42 (m, 2H), 1.22 (d, *J* = 6.6 Hz, 3H). ^13^C NMR (101 MHz, D_2_O) δ 173.7, 166.3, 151.8, 141.7, 102.7, 94.5 (d, *J* = 6.2 Hz), 88.5, 83.2 (d, *J* = 9.2 Hz), 73.8, 73.0, 70.8, 69.6 (d, *J* = 9.7 Hz), 64.9, 60.3, 55.0, 53.6 (d, *J* = 8.6 Hz), 42.0, 32.1, 18.6. The MS (ESI) value calculated for C_19_H_28_N_6_O_17_P_2_ (M‐H^+^) is 675.1, and the found value is 675.02 *m/z*.

^1^H, ^13^C and COSY NMR spectra are shown in Supplementary Figure 3.

## REAGENTS AND SOLUTIONS

*Use distilled, deionized water in all recipes*.

### Reaction buffer, 2×

40 µl of Tris·HCl buffer stock solution (1 M, pH 8)
4 µl of MgCl_2_ buffer stock solution (0.5 M)
40 µl of ATP stock solution (50 mM)
40 µl of UTP stock solution (50 mM)
20 µl of GlcNAc analogue stock solution (50 mM)
40 µl of BSA stock solution (10 mg/ml in PBS)
16 µl of milliQ water

Add all the reagents to a microcentrifuge tube and mix gently by inversion.

This is for a total volume of 200 µl; the volume can be scaled up accordingly.

Prepare directly before the reaction is set up and discard any leftovers at the end of the experiment.

### Sugar stain

200 µl 3‐methoxyphenol
5 ml sulfuric acid
194.8 ml ethanol

In a beaker, slowly add the acid to the ethanol, followed by 3‐methoxyphenol and mix them.

Transfer into an amber glass container with a lid.

Store at room temperature in a fume hood for up to several months.

Preparation of the stain should be carried out in a fume hood as the reagents are toxic, corrosive, and flammable.

## COMMENTARY

### Critical Parameters

#### Chemical synthesis of bioorthogonally tagged acylamide analogues of D‐GlcNAc and D‐GalNAc

Some reactions, depending on the bioorthogonal moiety, may require longer reaction times or a larger excess of the carboxylic acid. Other coupling reagents, such as HATU and COMU, can also be used instead of EDC and HOBT. In one case, for gram‐scale reactions, we have observed partial double incorporation of the bioorthogonal tag, most likely by ester bond formation with the anomeric hydroxyl group. In that case, the side reaction could not be prevented by changing the activators, temperature, reaction times, or equivalents of the acid. Crucially, we also observed that with small, branched azido acids such as L‐ or D‐azidoalanine, the reaction is sluggish and results in low yields. We observed that analogues containing small tags are difficult to obtain in high purity with good yields. In all of these cases, the issues were eliminated by using the Alternative Protocol.

The reaction can be scaled down or up, but care should be taken for potential side reactions and more challenging purifications, especially with small tags.

#### Conversion of D‐GlcNAc or D‐GalNAc analogues to UDP‐sugars on analytical‐ (reaction scouting) and preparative‐scale enzymatic synthesis and purification

We recommend the use of 2× Reaction buffer as it ensures reproducible results. Aliquoting reagents separately in small volumes in each reaction could lead to inconsistencies, resulting in irreproducible data. To obtain an accurate quantification of the turnover, all reaction and standard curve samples should be prepared, treated, and analyzed under the same conditions. Enzymes should be stored at −80°C as small single‐use aliquots to maintain consistency in their activity. Using enzyme aliquots with the same number of “freeze and thaw” cycles will allow for cross‐comparison between analogues and AGX1 variants. Removal of the enzymes before injecting them into the analytical column is highly recommended to prevent any column blockage and ensure sample reproducibility.

Keeping concentrations of substrates, ATP, and UTP as per the protocol when scaling down or up the analytical and/or preparative reaction is critical. We found that a further excess of these reagents reduces enzyme activity and decreases product formation.

Some GlcNAc/GalNAc analogues may never reach more than 30%–50% turnover to the UDP‐sugars with either of the AGX1 variants, despite increased reaction time and enzyme amount. Although not ideal, such turnover should still allow for obtaining reasonable amounts of the corresponding UDP‐GlcNAc/GalNAc analogues.

While the purification protocol allows for some alterations, the isolated product should be >95% pure. Prior to purification, the reaction mixture is treated with CIAP. We were able to purify some analogues without prior enzymatic digestion, while for others, CIAP treatment is essential to allow the isolation of the pure product. We recommend this treatment for all analogues since it is not labor‐intensive and aids purification even when not strictly necessary. Desalting of the dried reaction mixture before HPLC purification is crucial, as the reaction buffer has high salt content. Without desalting, the mixture would not be soluble in the small volume of eluent required for HPLC purifications. In this protocol, we performed desalting with solid‐phase extraction. Alternatively, size‐exclusion chromatography can also be used, although the procedure would require longer times.

During the characterization of UDP‐sugar analogues using ^1^H NMR, we observed that the peaks corresponding to vinylic protons in the uracil moiety (approximately *δ* = 7.85 and 5.95) gradually distributed into several peaks. Attempts to repurify compounds did not remove these additional peaks. Variable‐temperature NMR experiments were conducted, revealing that the additional peaks disappeared at elevated temperatures. We attribute this phenomenon to either rotamers or tautomers, for instance, of the uracil moiety (Delchev, 2010; Fedeles et al., 2022). More information can be found in our previously published work (Liu et al., 2024).

### Troubleshooting

Table 1

**Table 1:** Troubleshooting Guide for Chemoenzymatic Synthesis and Purification of UDP‐GlcNAc and UDP‐GalNAc Analogues

Problem	Possible cause	Solution
TLC not showing any product spots	Loading on TLC not sufficient	Repeat TLC, making sure you spot generously.
Incomplete reaction between the amino sugar and acid	Slower reaction Some reagents probably not added in adequate amounts Sugar not fully dissolved in methanol	Leave for longer times. Add more reagents. Add more triethylamine. Use the alternative method.
HPLC not yielding pure sugar analogue	Analogue very hydrophilic, eluting together with triethylamine	Use the alternative method.
Sample not showing the product of the enzymatic reaction during analytical UPLC‐MS	Missing substrate or reagent Inactive enzyme LC‐MS instrument problem	Check if substrate or reagents are added by looking at the expected ions. Check enzyme activity by testing AGX1 WT and GlcNAc and/or GalNAc. Check if the sample has been taken from the sample tray. Check for air bubbles at the bottom of the insert vial.
Preparative‐scale enzymatic reaction not proceeding as expected	Reagent concentration not optimal pH not optimal Enzyme not active	Check protocol. Check pH and adjust to pH 8 if needed. Check enzyme activity by testing AGX1 WT and GlcNAc and/or GalNAc.
CIAP treatment not resulting in complete hydrolysis of nucleotides	Not enough CIAP Longer time needed	Add more CIAP. Leave for longer.
Final UDP‐sugar analogue not pure after HPLC	CIAP treatment not effective. Faulty HPLC run	Identify impurities using LC‐MS and NMR. Depending on impurities, repeat CIAP treatment and repurify or directly repurify.

### Time Considerations

#### Basic Protocol 1

Chemical synthesis of bioorthogonally tagged acylamide analogues of D‐GlcNAc and D‐GalNAc can be completed within 2–5 days, depending on the reaction and the outcome of the first purification. On the first day, setting up the reaction and monitoring requires 1 to 2 hours. The time required for flash chromatography will depend on the type of system used. Overall, at least 1 day will be required to check fractions, dry, and characterize the product(s) using NMR. If a secondary reverse‐phase chromatography is required, an additional 2 or 3 days will be needed for purification, lyophilization, and product characterization. Larger‐scale reactions may require more time for solvent evaporation and lyophilization.

#### Alternate Protocol

Each reaction would require 1 to 3 days. This includes purification and characterization of the products.

#### Basic Protocol 2

Conversion of D‐GlcNAc or D‐GalNAc analogues to UDP‐sugars on analytical‐ (reaction scouting) and preparative‐scale enzymatic synthesis and purification can be completed within 5‐8 days including waiting time for data aquisition and lyophilization. Preparing stock solutions, setting up scouting reactions, and preparing samples for the LC‐MS analysis, including standard curve samples, will take a full day. Depending on the number of samples (analogues), 1 or 2 days will be required for running samples (data acquisition) and interpreting results. The preparative scale set up will take 1 h, but the reaction will proceed for 1 day, with an additional day required for the CIAP treatment. Quenching of the reaction, enzyme removal, and lyophilization of the crude residue will take 1 day. An additional 3 to 4 days will be required for desalting and HPLC purification. The actual purification step will require 1 to 2 h. The rest of the time will be used for data acquisition, data analysis, and drying the pure product by lyophilization.

### Author Contributions

**Benjamin Schumann**: Conceptualization; funding acquisition; writing—original draft; writing—review and editing. **Ganka Bineva‐Todd**: Data curation; formal analysis; writing—original draft; writing—review and editing.

### Conflict of Interest

The authors disclose no conflict of interest.

## Supporting information

Figures S1 to S3.

## Data Availability

Procedures discussed here were previously published (Liu et al., 2024). These reports contain detailed experimental and compound characterization procedures.
