# Supplementary material for: Chemoenzymatic Synthesis and Purification of Bioorthogonally Tagged UDP‐GlcNAc and UDP‐GalNAc Analogues
Source: Curr Protoc. 2025 Dec 17;5(12):e70277. doi: 10.1002/cpz1.70277 (PMC12710852; doi:10.1002/cpz1.70277)
Supplement: Supplementary file 1 — Figures S1 to S3. [file CPZ1-5-0-s001.pdf]

Chemo-enzymatic synthesis and purification of bioorthogonally tagged UDP-GlcNAc and UDP-GalNAc analogues

SUPPORTING INFORMATION

Ganka Bineva-Todd[a*], Benjamin Schumann[a,b,c*]

[a] Chemical Glycobiology Laboratory, The Francis Crick Institute, NW1 1AT London, U.K

[b] Department of Chemistry, Imperial College London, W12 0BZ, London, U.K.

[c] Faculty of Chemistry and Food Chemistry, TUD Dresden University of Technology, 01069, Dresden, Germany

*Correspondence: ganka.bineva@crick.ac.uk, Benjamin.schumann@tu-dresden.de

^{1}H NMR, D_2O, 400 MHz

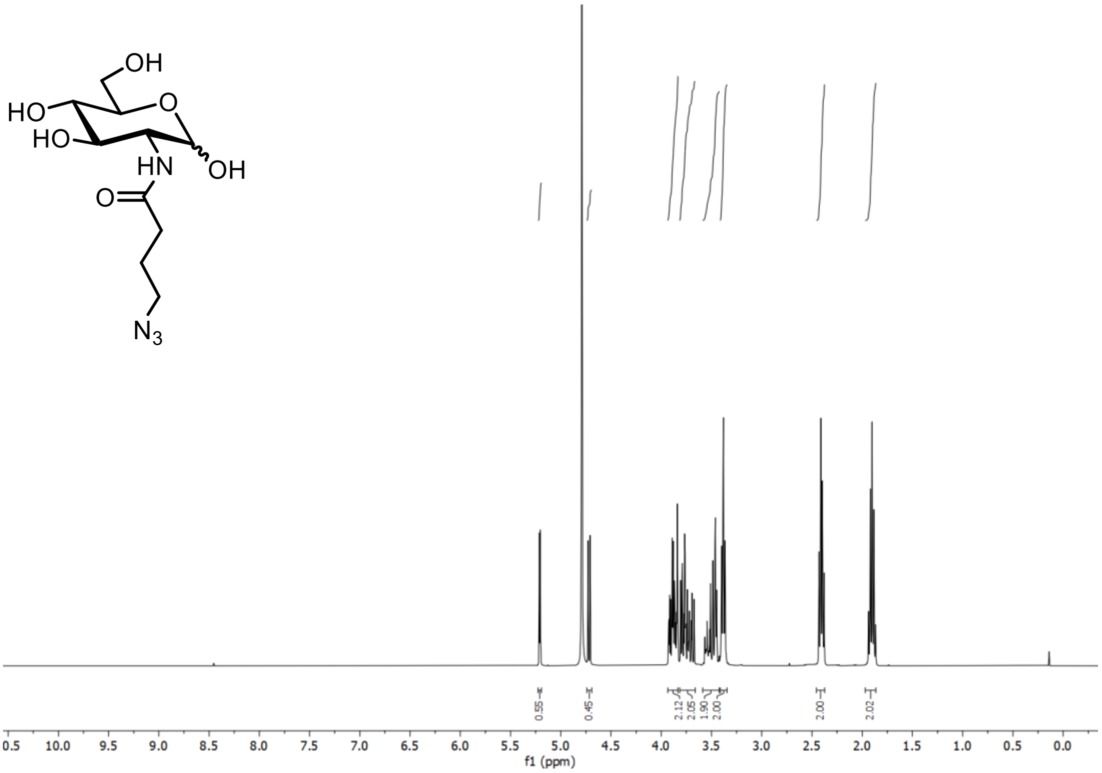

^{13}C NMR, D_2O, 400 MHz

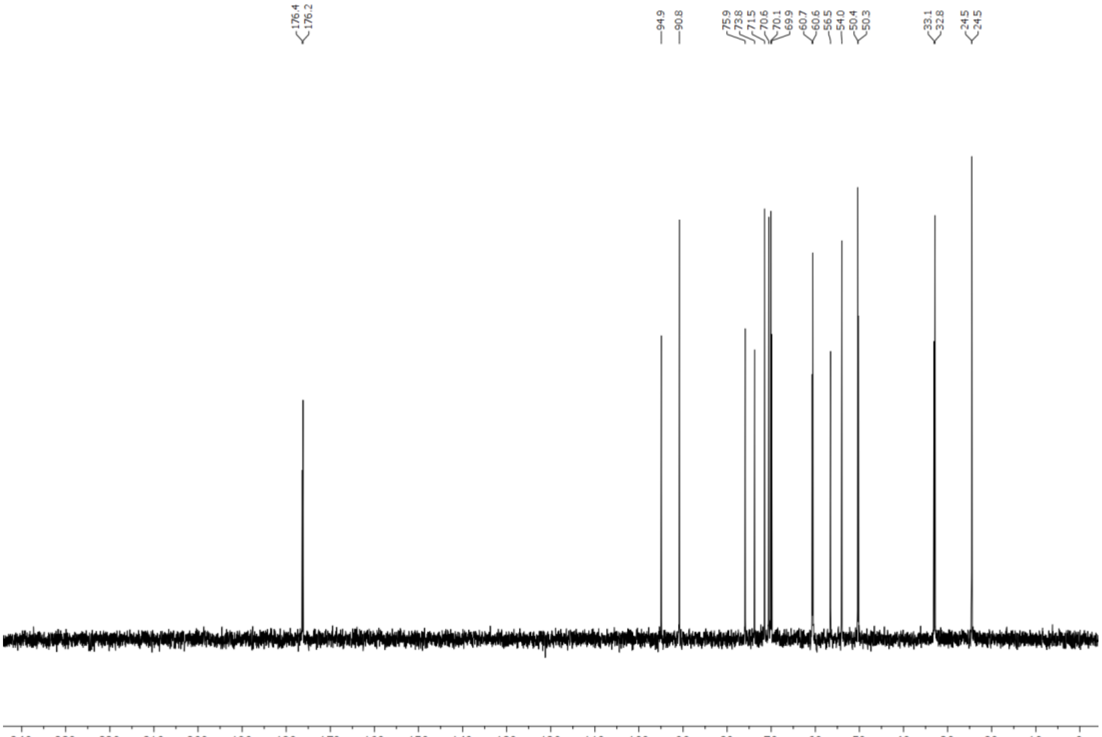

COSY, D_2O, 400 MHz

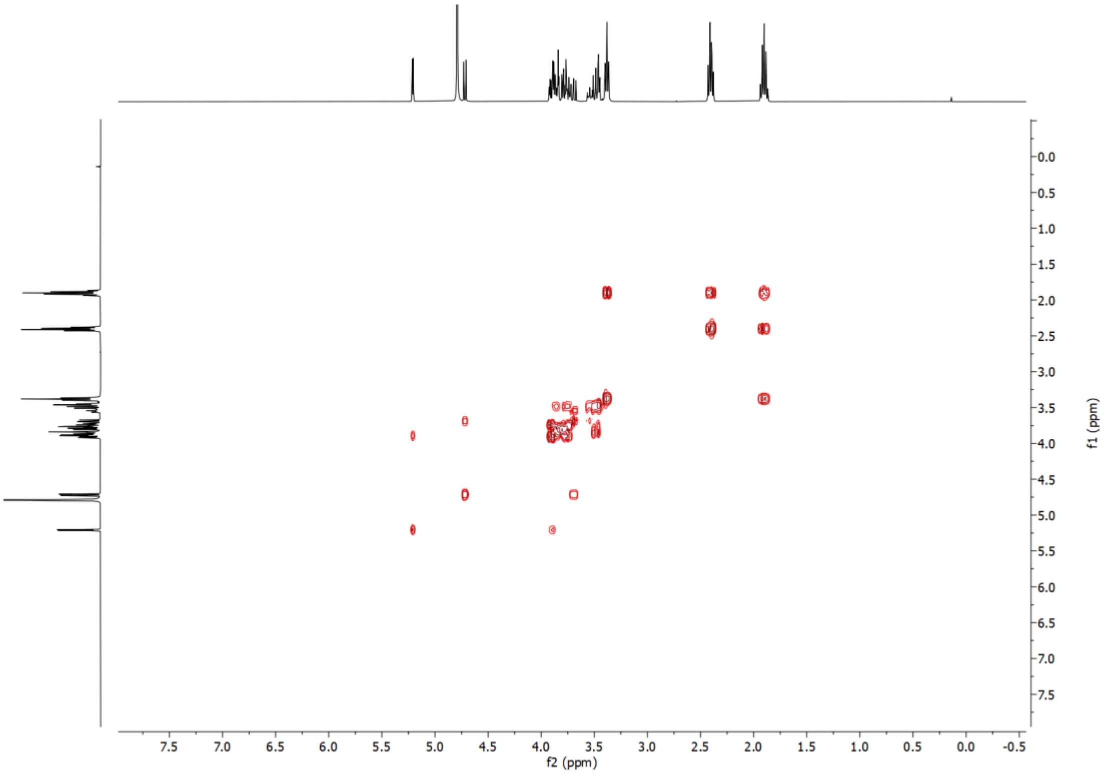

HSQC, D_2O, 400 MHz

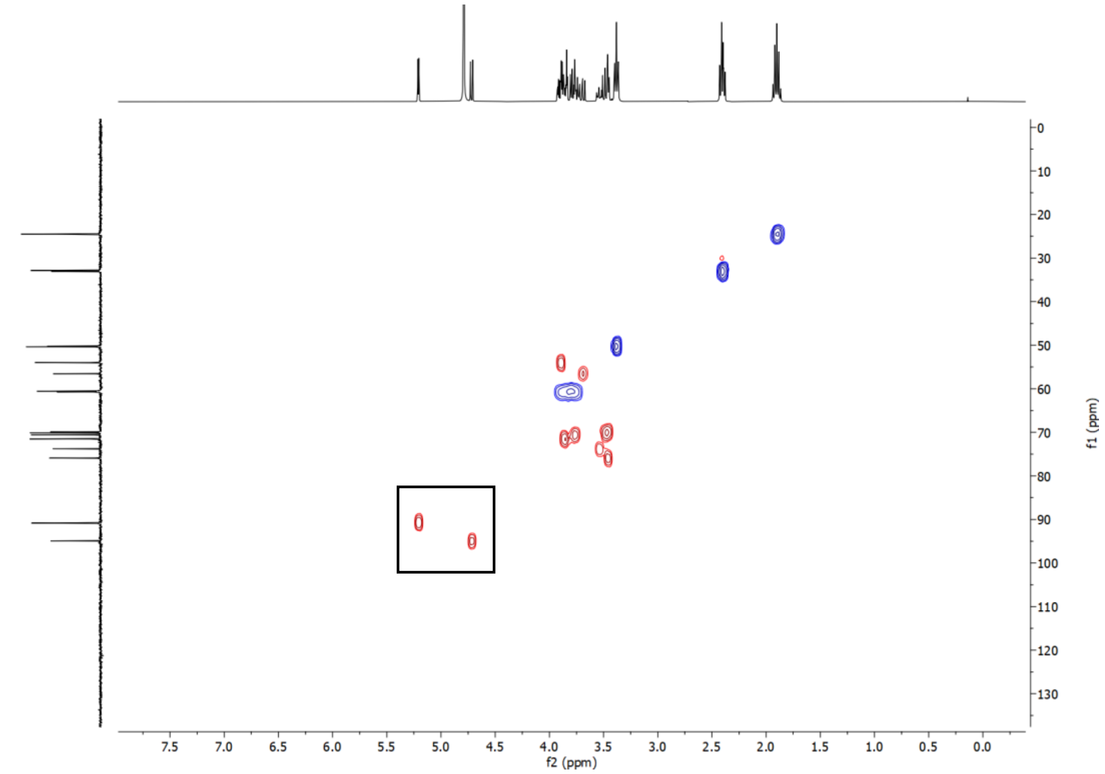

Supporting Fig. 1: Example of ^{1}H NMR, ^{13}C NMR, COSY and HSQC spectra of GlcNAc analogue (GlcNButAz) obtained via Basic Protocol 1. Spectra shows the product is an anomeric mixture indicated by the two doublets at 4.7 ppm and 5.2 ppm in the ^{1}H NMR and two singlets between 90

and 100 ppm in the ^{13}C spectra, also highlighted with square in the HSQC spectra. Data obtained with Bruker Avance 400 MHz spectrometer at 298 K.

A

^{1}H NMR, D_2O, 400 MHz

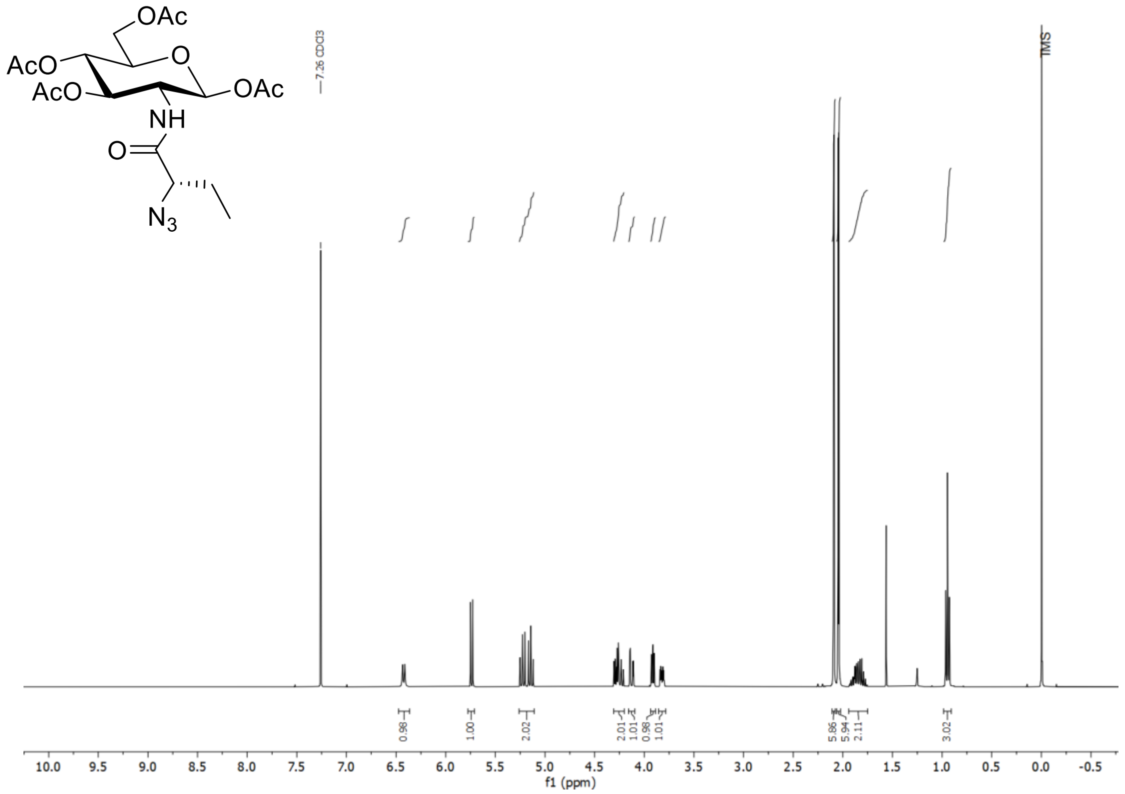

^{13}C NMR, D_2O, 400 MHz

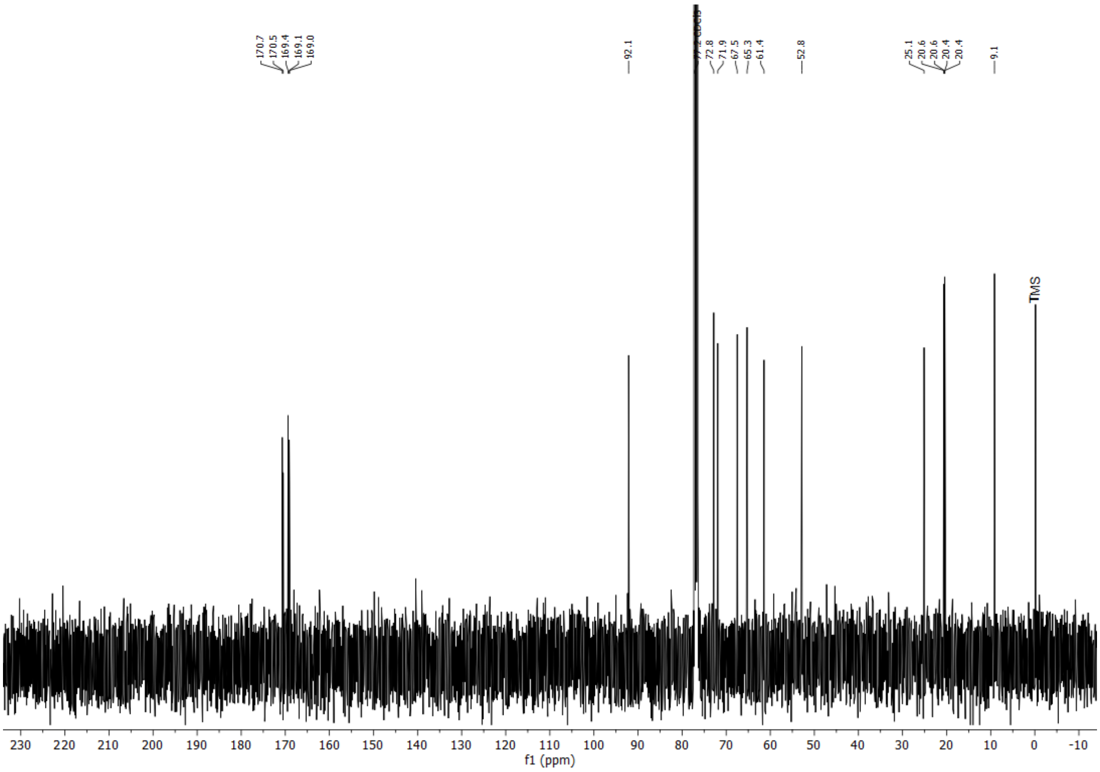

COSY, D_2O, 400 MHz

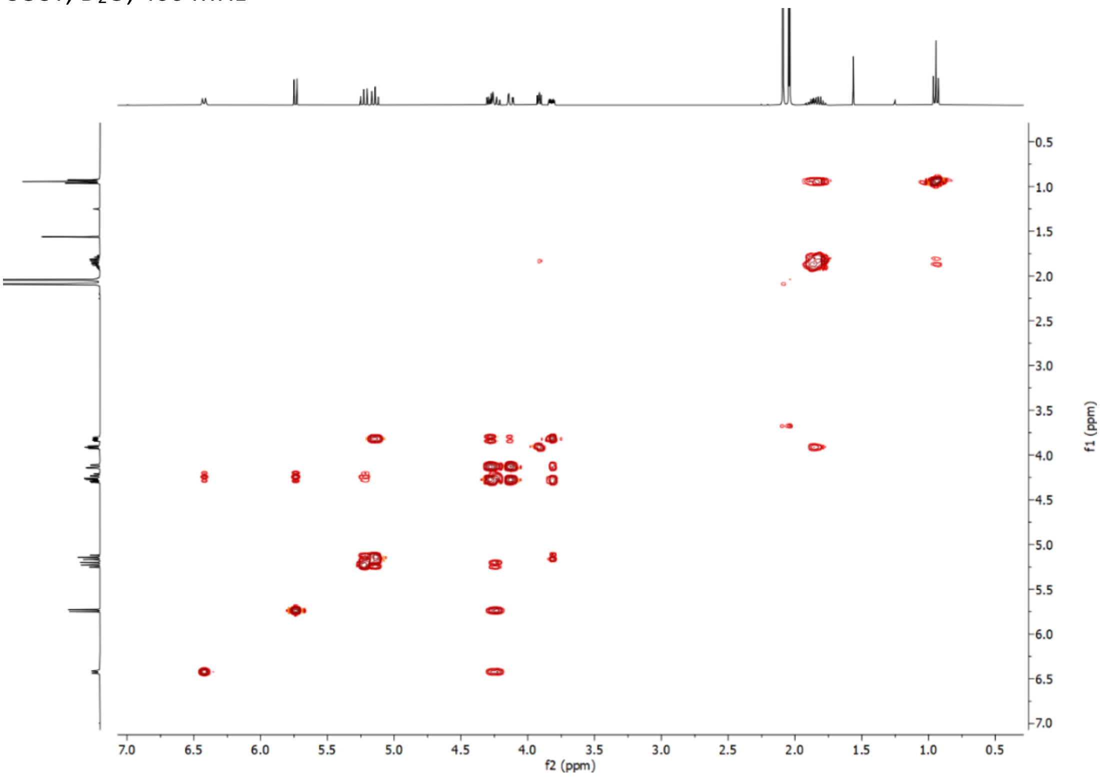

HSQC, D_2O, 400 MHz

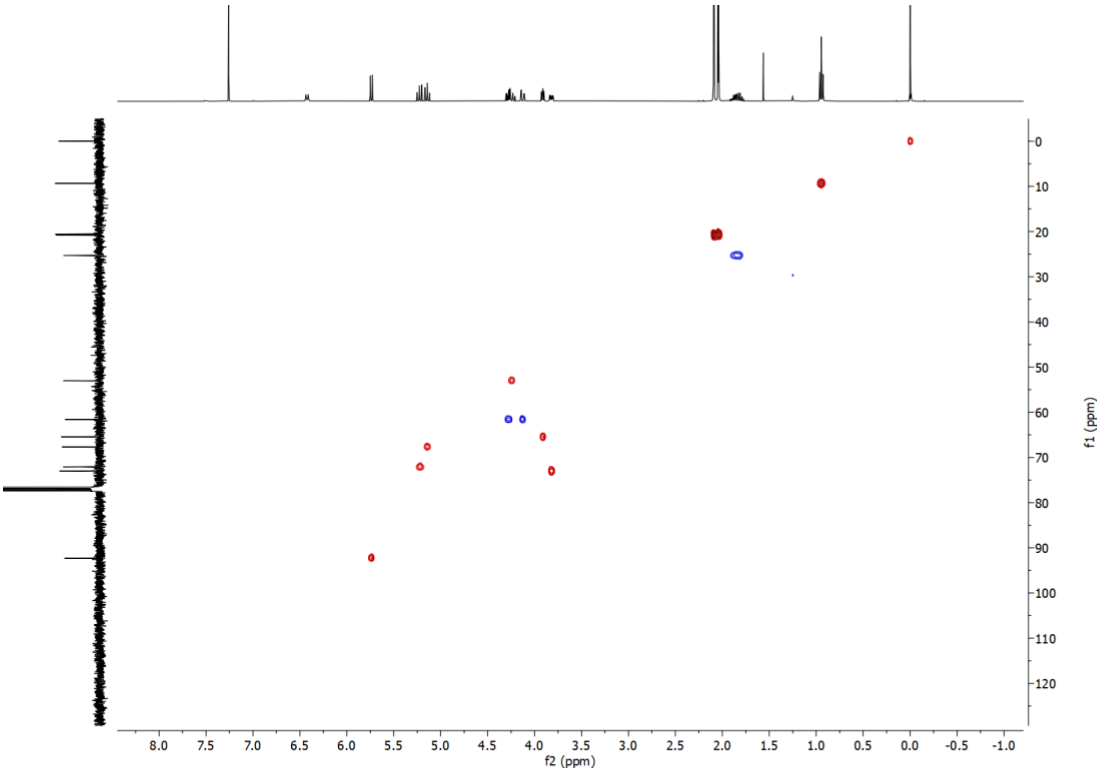

B

^1H NMR, D_2O, 400 MHz

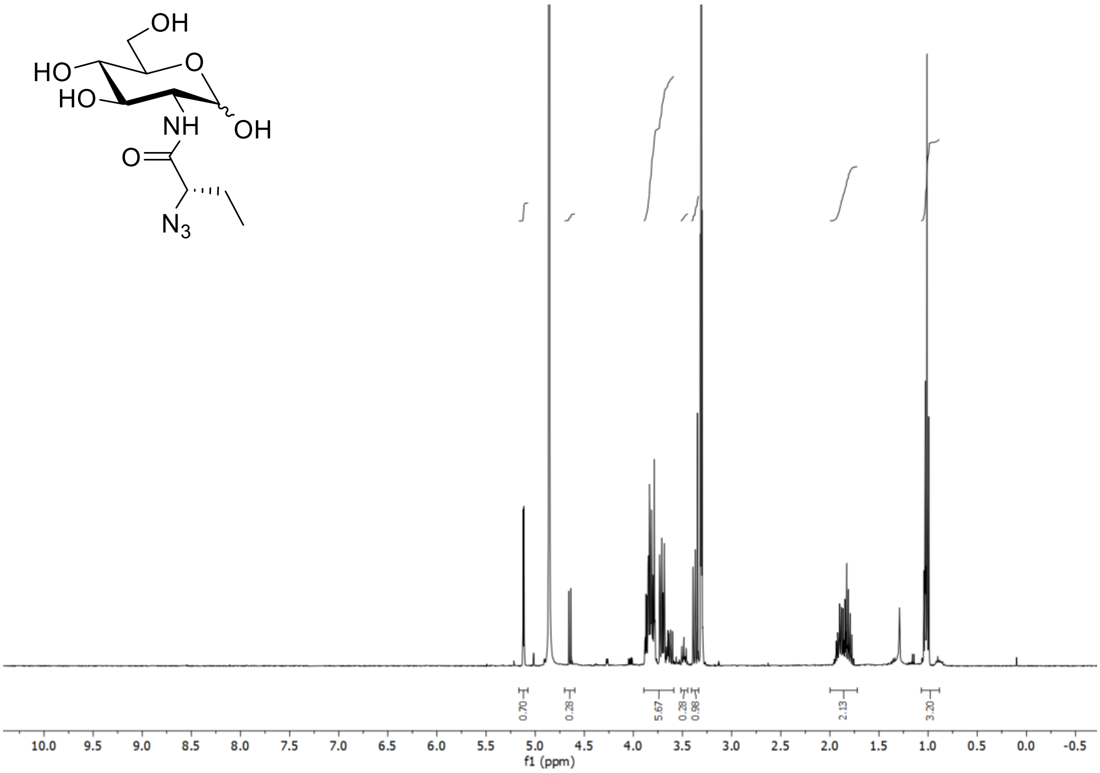

^{13}C NMR, D_2O, 400 MHz

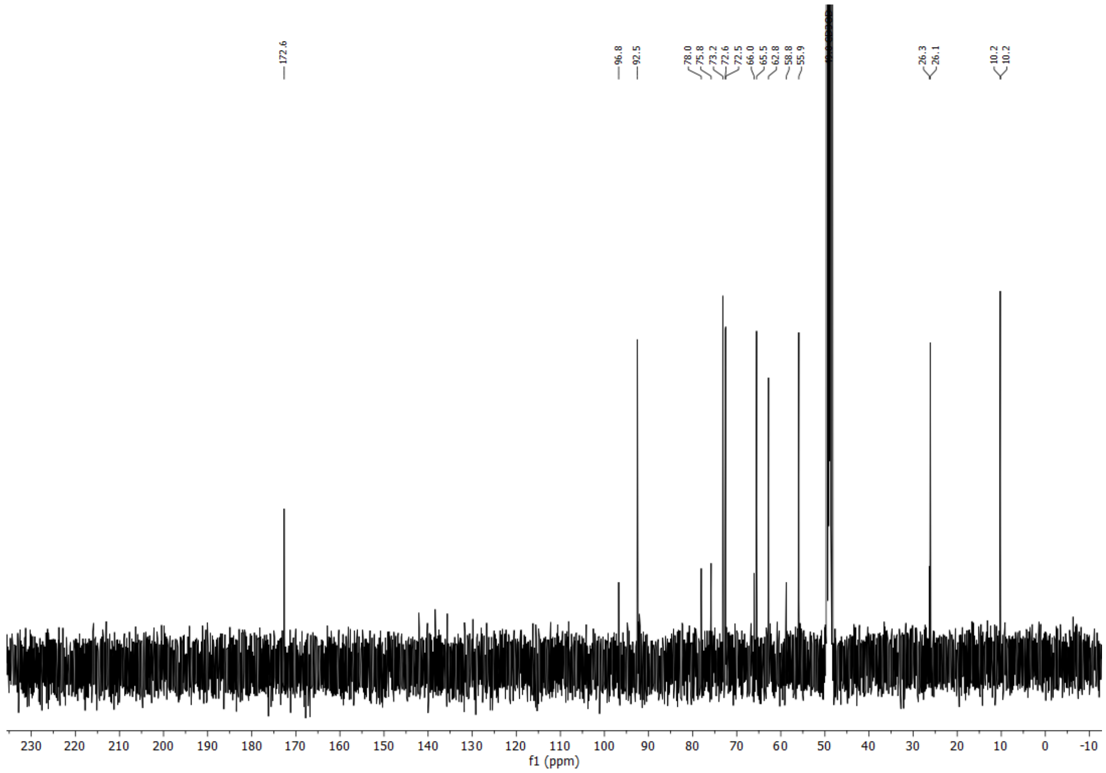

COSY, D_2O, 400 MHz

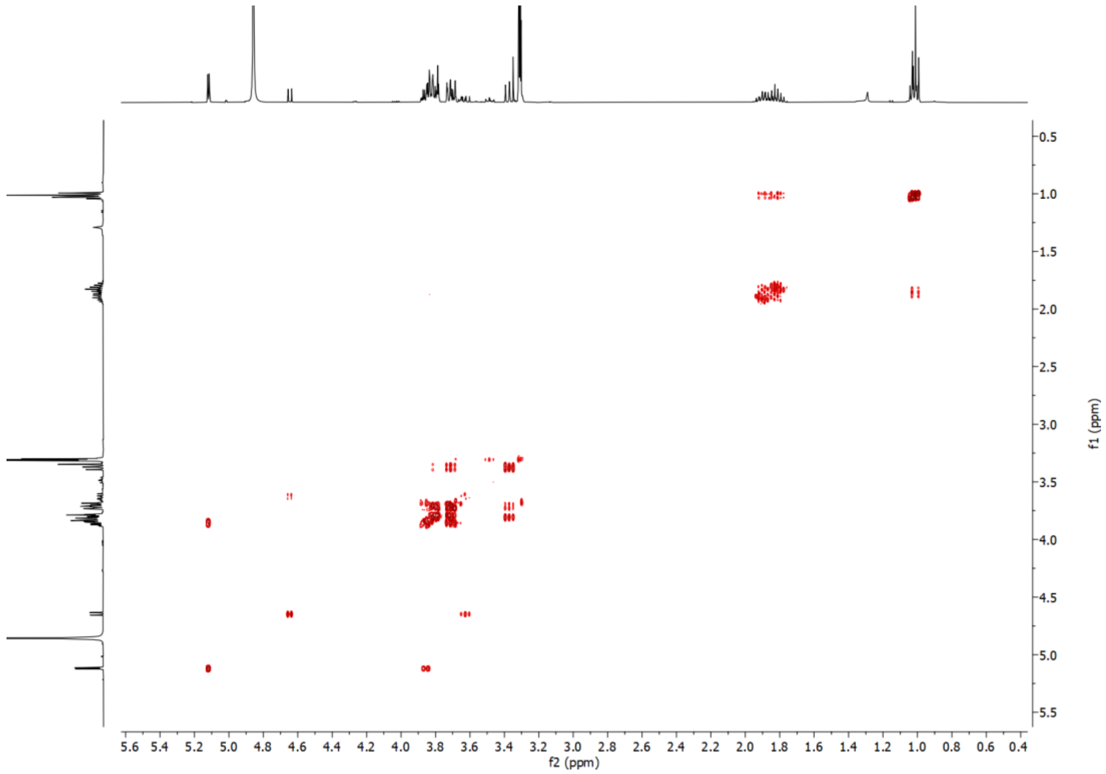

HSQC, D_2O, 400 MHz

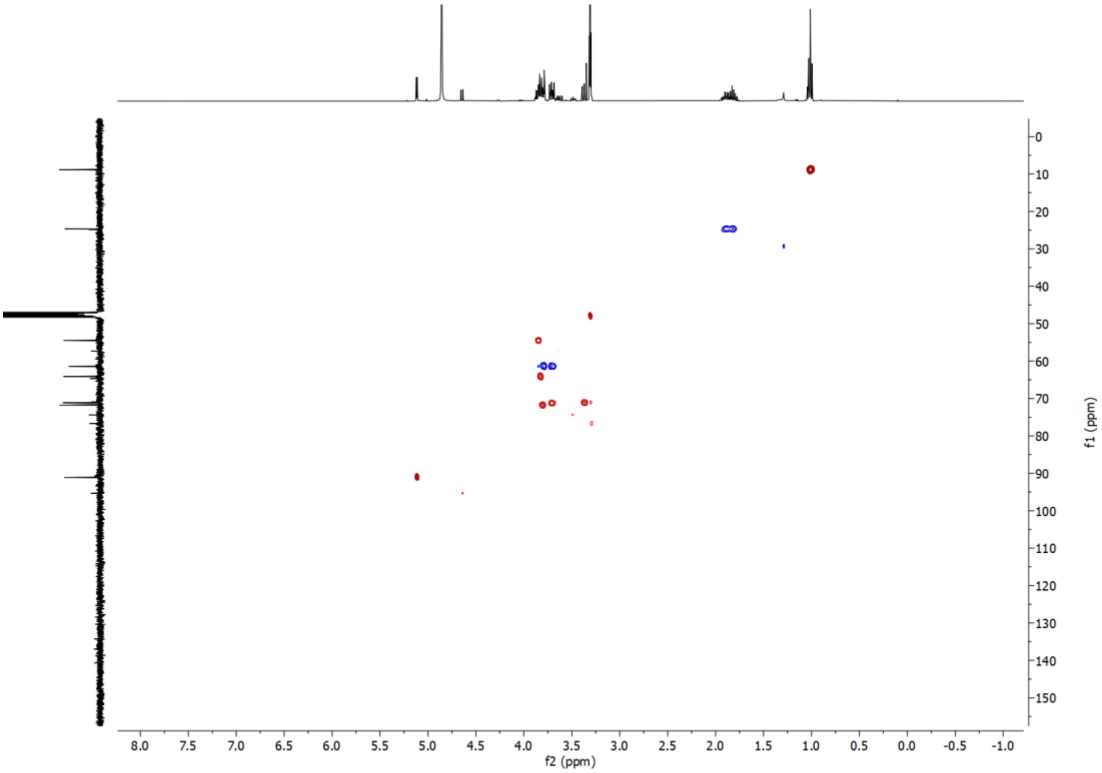

Supporting Fig. 2: Example of 1D and 2D NMR spectra of GlcNAc analogue obtained via Alternative Protocol 1. (**A**) ^{1}H NMR, ^{13}C NMR, COSY and HSQC spectra of the intermediate product Ac_4GlcNEtAz(*S*) after the coupling reaction. (**B**) ^{1}H NMR, ^{13}C NMR, COSY and HSQC spectra of the final product GlcNEtAz(*S*) after the deprotection reaction. Data obtained with Bruker Avance 400 MHz spectrometer at 298 K.

^{1}H NMR, D_2O, 400 MHz

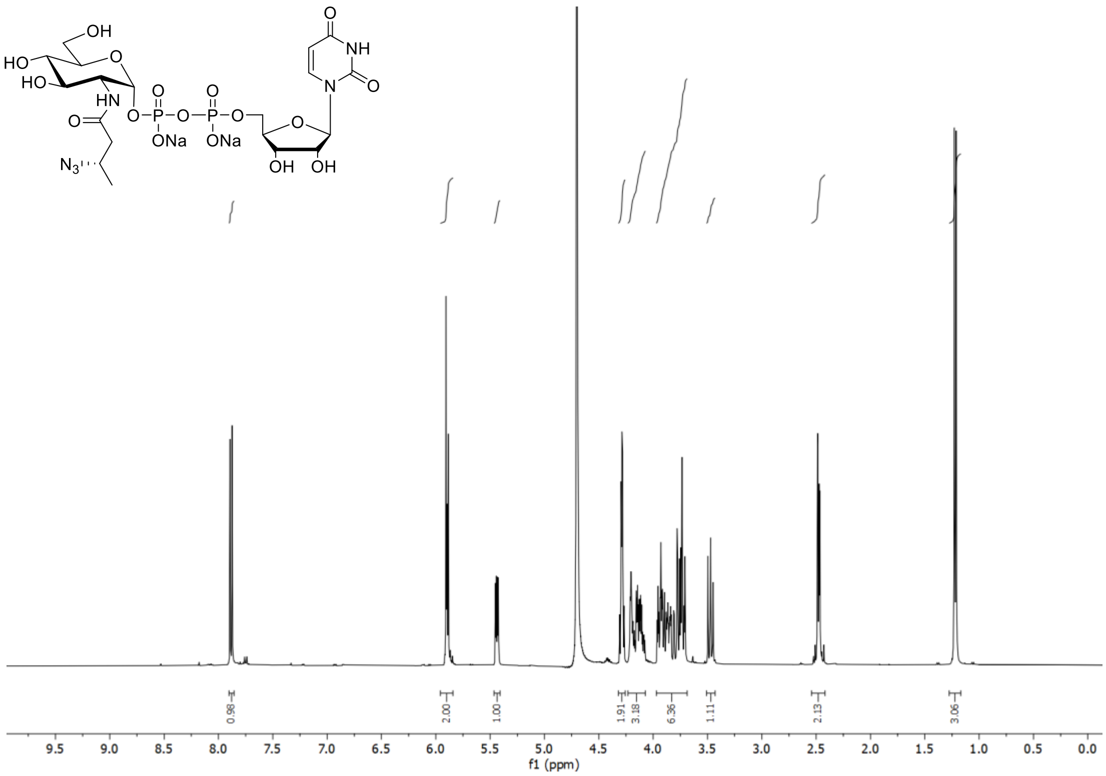

^{13}C NMR, D_2O, 400 MHz

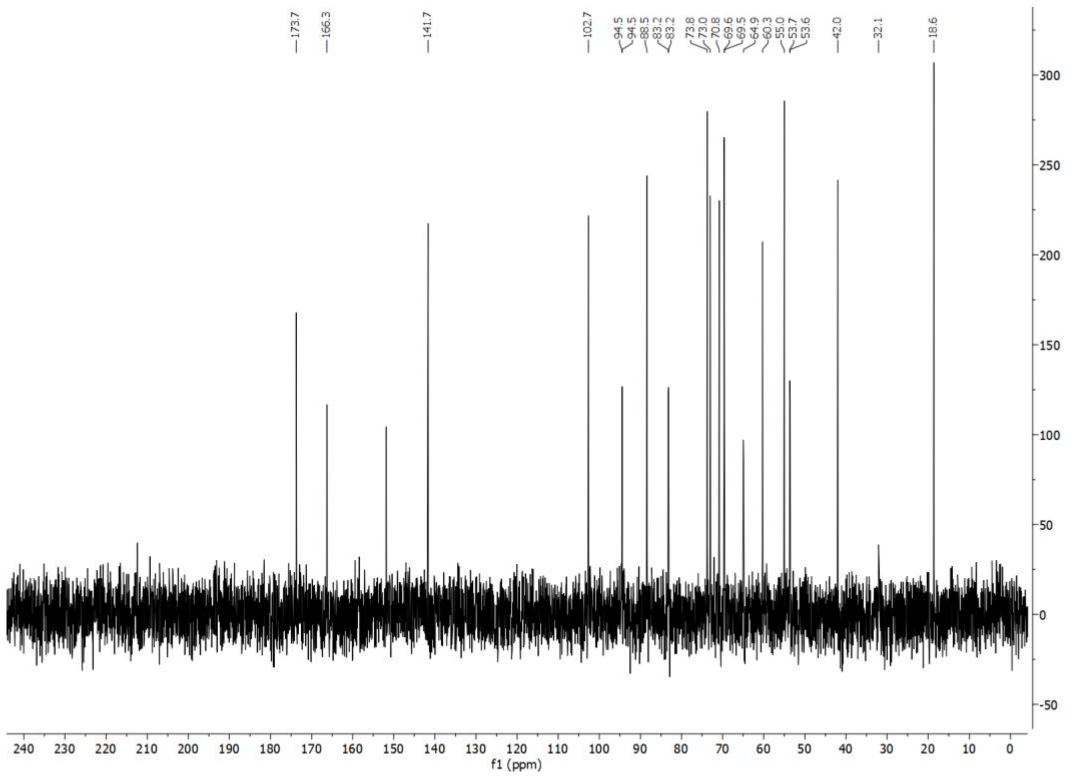

HSQC, D_2O, 400 MHz

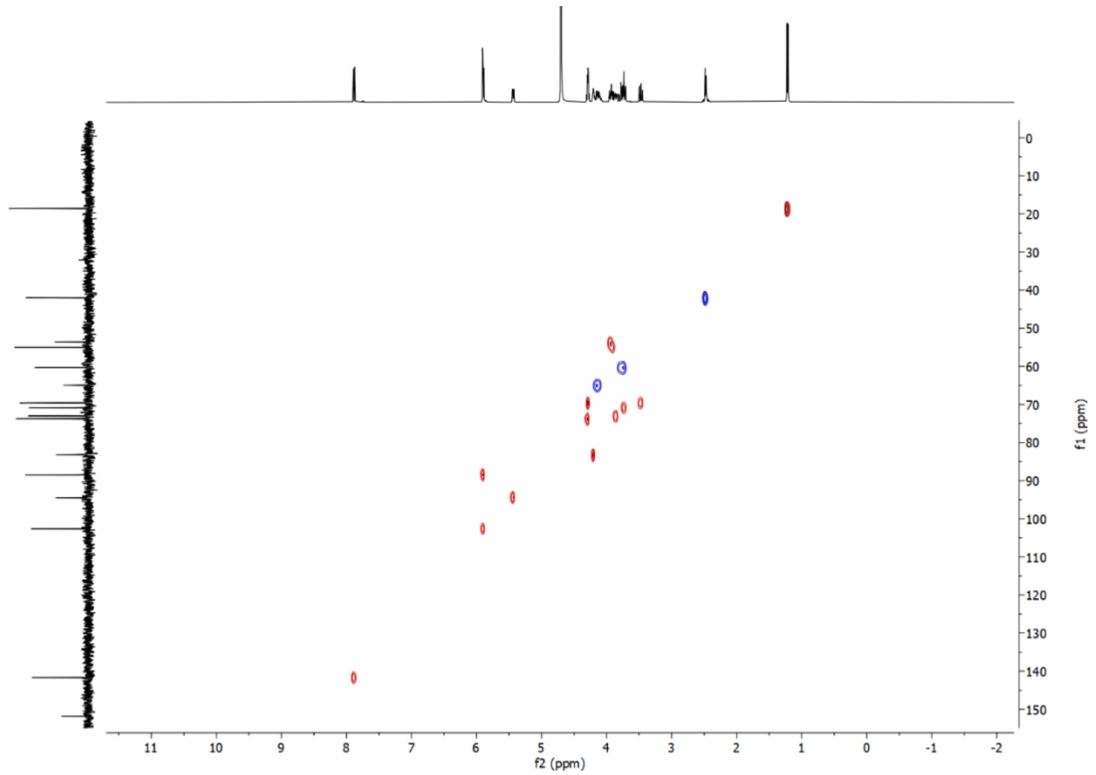

Supporting Fig. 3: ^{1}H NMR, ^{13}C NMR and HSQC spectra of the final analogue UDP- GlcNPrAz3Me(*S*). Data obtained with Bruker Avance 400 MHz spectrometer at 298 K.
